# The Influence of Urbanism and Information Consumption on Political Dimensions of Social Capital: *Exploratory Study of the Localities Adjacent to the Core City from Brașov Metropolitan Area*, *Romania*

**DOI:** 10.1371/journal.pone.0144485

**Published:** 2016-01-25

**Authors:** Cătălina-Ionela Rezeanu, Arabela Briciu, Victor Briciu, Angela Repanovici, Claudiu Coman

**Affiliations:** 1 Department of Sociology, Faculty of Sociology and Social Work, University of Bucharest, Bucharest, Romania; 2 Department of Social Sciences and Communication, Faculty of Sociology and Communication, Transilvania University of Braşov, Brașov, Romania; 3 Department of Faculty of Product Design and Environment, Transilvania University of Braşov, Brașov, Romania; Tianjin University of Technology, CHINA

## Abstract

**Background:**

The last two decades have seen a growing trend towards the research of voting behavior in post-communist countries. Urban sociology theorists state that not only space structures influence political participation, but also space structures are changing under the influence of global, local, and individual factors. The growing role played by information in the globalised world has accelerated the paradigm shift in urban sociology: from *central place model* (based on urban-rural distinction and on monocentric metropolitan areas) to *network society* (based on space of flows and polycentric metropolitan areas). However, recent studies have mainly focused on countries with solid democracies, rather than on former communist countries. The present study aims to analyze the extent to which a new emerging spatial structure can be envisaged within a metropolitan area of Romania and its consequences for the political dimensions of social capital.

**Methods:**

The Transilvania University Ethics Commission approved this study (S1 Aprouval). The research is based upon individual and aggregate empirical data, collected from the areas adjacent to the core city in Brașov metropolitan area. Individual data has been collected during October 2012, using the oral survey technique (S1 Survey), based on a standardized questionnaire (stratified simple random sample, N = 600). The National Institute of Statistics and the Electoral Register provided the aggregate data per locality. Unvaried and multivariate analyses (hierarchical regression method) were conducted based on these data.

**Results:**

Some dimensions of urbanism, identified as predictors of the political dimensions of social capital, suggest that the area under analysis has a predominantly monocentric character, where the rural-urban distinction continues to remain relevant. There are also arguments favoring the dissolution of the rural-urban distinction and the emergence of polycentric spatial structures. The presence of some influences related to the information consumption on all six indicators of the political dimensions of social capital under analysis suggests the occurrence of emerging forms of a space of flows. The identified effects of social problems associated with transport infrastructure and of migration experience on the political dimensions of social capital, also support the emergence of space of flows.

**Conclusions:**

We recommend that, in the urban studies in former communist countries, conceptualization of urbanism as predictor of the political dimensions of social capital should consider both the material dimensions of space, as well as the dimensions of information consumption and migration experience.

## Introduction

Over the past 70 years, social scientists from different schools of thought (the Columbia studies, the Michigan model, the American Voter model, the funnel of causality, the rational choice model etc.) have been trying to explain voting behavior [1]. The last two decades have seen a growing trend towards the research of electoral behavior in post-communist countries [2–4]. On the one hand, scholars studying social capital argue increasingly about the dissolution of trust and civic engagement in post-communist countries [5, 6]. On the other hand, urban sociology theorists point out that each major politico-economic transformation leads to spatial transformations, which influence social relations and social actions [7]. Up to now, the studies on voting behavior have largely disregarded the impact of recent spatial transformations. Therefore, the main purpose of this article is to demonstrate the applicability of some theoretical models from urban sociology to study voting behavior in a recently created metropolitan area from post-communist Romania. We argue that the study of the political dimensions of social capital (voter turnout, trust in politicians and politicians’ name recognition) in post-communist countries could be furthered by taking into account the ongoing changes of spatial structures.

### Voting behavior, social capital and political participation

The sociological study of voting behavior focuses on “the way individuals obtain, select, and process information related to the political arena; the various forces that shape this process; the relevance individuals attribute to the political sphere; and how they decide to participate in or refrain from specific political actions”[8].

Some dimensions of voting behavior have been measured as dimensions of social capital. Despite the popularity of the concept of social capital in the social sciences, to date, there has been little agreement on its definition and measure. Some of the most cited definitions are those formulated by Bourdieu [9], Coleman [10], Fukuyama [11], and Putnam [12], the last one focusing on the role of civic engagement (political participation, volunteering), the norms of social networks (reciprocity), and trust (in people and public institutions). This perspective was adopted by OECD [13] measuring social capital based on four dimensions (personal relationships, social network support, civic engagement, and trust and cooperative norms), which were simplified by the Office for National Statistics from the UK [14] reducing them to three (trust, participation, and networks). More complex measures are used by The World Bank [15] based on five dimensions of social capital: groups and networks, trust, collective action, social inclusion, information and communication; and by the Australian Bureau of Statistics [16] based on six dimensions: support structures, social participation, trust in people and institutions, tolerance, altruism and voluntary work. Because all of the presented models have in common trust and civic engagement as dimensions of social capital, we decided to focus on these dimensions of social capital. Moreover, the authors of a recent Romanian study [17] argued that trust (including trust in political institutions) is a pre-condition of political participation, which can be characterized by interests in politics and voter turnout.

The relation between spatial arrangements and social capital has been thoroughly studied in the literature [18–21], as well as the relation between social capital and the use of specific information means [22–27]. However, there are no studies from post-communist countries, including integrated analyses of the effects of spatial arrangements, information consumption, and political dimensions of social capital. We believe that Romania represents an adequate context for studying the determinants of the political dimensions of social capital mainly because the country experienced one of the harshest political régimes characterized by a high level of repression. According to a local study [17], in Romania, from 1990 to 2014 the trust in the institutions of the political system showed a decreasing trend, and so did the voter turnout, while the interest in politics was found to be relatively low in 2012.

We started from a premise, inspired by ideas from urban sociology, that the citizen’s political participation is influenced by the spatial characteristics of the locality they live in and by their individual and aggregate actions of information consumption. The first influence was derived from the concept of urbanism as a way of life while the second one is predicated on Castell’s model of the space of flows. These models will be explained as follows.

### From rural-urban distinction to metropolitan areas and space of flows

In its beginnings, the sociological study of space has played an important role in understanding the changes brought about by capitalism and urbanization (the migration of population from rural to urban settlements and the development of modern cities). The concept of urbanism (measured by size, density, and heterogeneity of urban population) was introduced into urban sociology in order to illustrate the impact of urban space on social relations and social practices. Based on the assumption that urbanization influences social relations, summarized by the concept of **urbanism as a way of life**, the classical studies of Wirth [28], consistent with the theories of Simmel [29], showed that living in the city leads to a decrease in sociability. The study concluded that, by separating the individual from his rural traditional community, the urban context of living brings isolation, alienation, individualism, and failure to accept social norms. This conclusion has been either invalidated [30], developed [31], or partially confirmed [32, 33]. The research findings into the negative impact of urbanism on social relations have been inconsistent and contradictory. The lack of consensus regarding the positive or negative effects of urbanism could be attributed to the fact that they were measured mostly in the context of the private sphere of life (personal and family relations), ignoring its possible effects in the public sphere of life (for instance, on voter turnout, trust in politicians and politicians’ name recognition).

Later on, as a result of the intensification of urbanization, the **distinction between rural and urban** faded, theoreticians estimating that the world will become urban in the future [7, 34]. Since the conceptualization of urban, as opposed to the rural, was viewed as an ideological construct, recommendations have been made that the studies in urban sociology should shift from the typology of spatial settlements to the analysis of contextual differences of socio-spatial processes (urbanization, suburbanization, formation of metropolitan areas etc.) [35], especially in former communist countries [36]. We considered Romania to be a proper context for such studies because of its spatial and political dynamics. The country shifted in 1989 from a planned economy to a market economy, and in 2007 joined the European Union. While in 1948 only 23% of the Romanian population lived in urban areas, after the communist period, the percentage reached 54% [37].In light of the recent theorizations from the literature, we considered that more studies are needed to analyze the extent to which the distinction rural-urban remained relevant in Romania during the transition period to the neoliberal economy and global capitalism.

The different stages of development of capitalism are reflected in different stages of urbanization, from the industrial city to metropolitan city and suburbs, and more recently to monocentric and polycentric metropolitan regions [38]. At the international level, the last decades have seen a growing trend towards studying the characteristics of metropolitan areas. In **monocentric** metropolitan areas, density decreases with the increase of the distance to the center of the core city, which concentrates the main economic functions of the region and which is the focal point of the radial transport network; in **polycentric** metropolitan areas, the key functions of the region are dispersed in several localities, which leads to the increase of population density and to the development of complex networks of transport infrastructure [39,40]. These new spatial structures depend on the size of the population and the distance to the center of the core city [41]. Despite the increasing interest of the researchers for the typologies of metropolitan areas, the investigation of this topic in post-communist countries is still scarce. We assumed that Romania in general and the metropolitan area of Brașov, in particular, would be a proper context for such studies. In Romania, around 7.4 mil people, meaning 34% of the population, live in metropolitan areas [42]. The most researched metropolitan area in Romania has been Bucharest area, the capital of the country [43–47]. While being located in the center of the country, Brașov metropolitan area, with Brașov as core city functioning as the connection node in internal and international transport infrastructure, has been poorly studied by researchers in the field of urban sociology. Brașov metropolitan area unfolds over a land area of more than 1700 km^2^, with a population exceeding 400,000 inhabitants, and includes 17 localities (3 municipalities, 4 cities, and 10 villages). According to an Epson Report, in 2005, Brașov County was included under the category of monocentric spatial structures [48]. Yet, the study was conducted 10 years ago, and considering the imperative of change in the countries and regions in transition to the market economy, we could assume that some transformations might have happened since then.

One major problem of polycentric metropolitan areas, especially in the post-communist countries, is the phenomenon of **urban sprawl**. During the socialist era, urban planning aimed to compact settlements. During the post-socialist era, in Central and Eastern Europe, due to the chaotic land restitution, the suburban sprawl intensified, leading to negative effects, such as isolation, residential segregation [49] and decreased quality of life caused by the lack of access to proper public service infrastructure [50]. This phenomenon was highlighted in Romania by geographic studies conducted both at the national level [51] and across Brașov metropolitan region [52, 53]. The preponderance of the urban sprawl phenomenon brings into the spotlight the importance of several new indicators that are used in measuring the concept of urbanism: the density of population and dwellings, the access to public utilities, public services, and transport infrastructure. Moreover, these studies focused mainly on describing and measuring the scale of the phenomenon and its impact on the quality of life without highlighting its consequences on social actions (like voting behavior).

Presently, more and more theories and studies in the field of urban sociology are shifting the focus from the research of material space to the research of informational space. The **space of flows** model, developed by Castells [54], analyses the impact of the globalization process on space, which shifts from a geographically well-defined local space to a space of multiple flows of capital, information, interactions, symbols, and technologies. The space of flow is linked to the development of cross-national hierarchies of cities, which act as strategic nodes on the information market [55]. Thus, in the new global society, mobility, transport infrastructure and access to information networks becomes more important than the static and local characteristics of the spatial settlements.

### Summary of research questions and objectives

Applying these premises to the present research, monocentric or polycentric spatial structure could be identified by researching the impact of material space on the political dimensions of social capital, and the space of flow by researching the impact of informational space on the political dimensions of social capital. We based our investigation on two research questions: what are the characteristics of the spatial structures of the studied area? How do they influence the political dimensions of social capital? To answer these questions, we formulated some objectives aiming to identify: 1) the characteristics of urbanism, information consumption and political participation in the studied area; 2) the impact of some indicators of urbanism (distance from the respondent's house to the center of Brașov Municipality and the urban administrative status of the locality) on other indicators of urbanism (like population density, access to public services and to public utilities); 3) the indicators of urbanism which have a significant impact on the indicators of political dimensions of social capital; 4) the indicators of information consumption which have a significant impact on the indicators of political dimensions of social capital; 5) the direction and intensity of these influences (S1 Survey).

## Materials and Methods

### Methods and techniques of data collection

This study is a secondary analysis of individual and aggregate data. Individual data were collected by using a standardized questionnaire. We conducted the research in October 2012 in the localities adjacent to the core city within Brașov metropolitan area, at the respondents' homes, using 17 operators and we centralized the data using IBM SPSS Statistics, version 20. One major drawback of this approach is that the indicators from the database were limited to the objectives of the primary study. In order to compensate this deficiency, we supplemented the database with aggregate indicators obtained from the National Institute of Statistics, and the National Electoral Register. The research database and the questionnaire are in the public domain, archived in the Transilvania University repository at http://aspeckt.unitbv.ro/jspui/handle/123456789/1863

### Ethics statements

The Transilvania University Ethics Commission approved the study (S1 Aprouval). The primary research has been conducted in cooperation with the County Council of Brașov, which endorsed the objectives and the methodology, and to make public the database and the analysis results. The informed consent was given orally. The potential respondent was informed about the objectives of the study before consenting to respond and was offered guarantees regarding the confidentiality of their personal data. The rationale behind choosing oral consent is that the Romanian institution of opinion polling is relatively new, and respondents are very reluctant to provide any signature for their responses. In order to preserve their anonymity, the personal data of the respondents (name and address) have been deleted from the database. To avoid any discrimination, the nationality variable was measured by an open question (What is your nationality?), followed later by coding the responses in two categories ("Romanian" and "Other nationality"). A similar approach was used for the age variable (which was not coded). Note that in conducting the research and publication of results, both local [56] and international ethical standards were followed. The results of the study, even though they came into possession of the County Council in 2013, were not publicly disclosed, nor were they subject of any scientific article so far.

### Selection of respondents

The research population consists of un-institutionalized citizens of Braşov metropolitan area, aged 18 and above. Around 90% of the inhabitants of Brașov metropolitan area live in urban localities [53]. Within Brașov metropolitan area, we focused on the localities adjacent to Braşov Municipality, where the urbanization rate reaches 22%.

A *stratified simple random sample* [57] was used, the strata being the localities adjacent to the core city in Brașov metropolitan area (15 localities: 2 municipalities, 4 towns, and 9 communes). The size of sub-samples for each stratum was set proportionally with the size of the population of the locality. The selection procedure was the unrepeated extraction by means of a random number table. For a sampling frame, we used the voting lists available at the polling stations in the localities under analysis. Assuming a 95% confidence level and a margin of acceptable error of 4%, the sample size was 600 respondents, stratified as follows: 205 respondents for the two municipalities, 188 for the 4 cities and 207 for the 10 villages. With respect to the socioeconomic profile of the respondents from the sample, 49% are male and 51% are female (which is the profile of Brașov County). The average age is 50 years and the average household income is 1536 RON (the equivalent of 347 Euro), respondents considering themselves poor rather than rich. In terms of education level, half of the respondents have secondary studies, 29% have higher education studies and 21% have primary studies. More than half are married, 11% belong to a nationality other than the majority one (Romanian), and 9% have at least one member of the family working abroad.

### Measurement

Four concepts have been measured: social capital (SC), urbanism (U), information consumption (IC) and socioeconomic status (SES). Although the database included a much larger number of variables, the scheme presented below includes only variables significantly correlated with indicators of the political dimensions of social capital (*r* > .01; *Sig*. < .05; P = 95%) (see Table A in S3 Appendix). In addition to the individual data collected by oral survey, a number of aggregate variables (at the level of locality) were recorded in the database, obtained from the National Institute of Statistics, via the Tempo online database [58], and from the national Electoral Register under the Permanent Electoral Authority [59–61] (see Table 1).

**Table 1:** Source of aggregate data.

Variable in the study	Original variable the database or the formula for the computation of the original variables	Database source
SC2	*PRESENCE*	PEA, Local Elections 2012
SC3	*PRESENCE*	PEA, Romanian Parliament Elections 2012
SC4	*PRESENCE*	PEA, Referendum for dismissal of the President of Romania 2012
U1	*AGR*101*B*	NIS, Tempo online database, year 2011
U2	*POP*101*D / AGR*101*B*	NIS, Tempo online database, year 2011
U3	*AGR*101*B / LOC*101*B*	NIS, Tempo online database, year 2011
U4	*LOC*103*B / POP*101*D*	NIS, Tempo online database, year 2011
U13	*SCL*101*C + SAN*1014 *+ ART*101*B*	NIS, Tempo online database, year 2011
U14	*GOS*106*B + GOS*110A *+ GOS*116*A*	NIS, Tempo online database, year 2011
U15	*ART*101*B*	NIS, Tempo online database, year 2011
U14/U1	(*GOS*106*B + GOS*110A *+ GOS*116*A*) / *AGR*101*B*	NIS, Tempo online database, year 2011
IC9	*ART*107*B*	NIS, Tempo online database, year 2011
IC9/U15	*ART*107A */ ART*101*B*	NIS, Tempo online database, year 2011

#### Contextual factors in post-communist countries

We appreciated that the conceptualizations used in the urban sociology should be supplemented by taking into account some specific features of post-communist countries, stated in the politico-economical literature [4]. First, in the post-communist countries, the inherited economic and political conditions might have influenced the voting behavior in the transition period, causing lack of trust in politicians, raise of the importance of politician’s name recognition and extreme variations in voter turnout. We included these indicators in the measurement of the political dimensions of social capital. Second, the transition to democracy and market economy involves politico-economical reforms, putting on political agendas issues imposed by international trends related to modernization, transport infrastructure, environmental conservation, and mobility. Because they change the spatial structure of localities and metropolitan areas, we used them as indicators of urbanism. Third, in post-communist countries the privatization process included the liberalization of mass media, whose specificity should be taken into account in measuring information consumption in Romania.

One of the most radical transformations occurred in the field of news, which evolved from the positively biased news presented in the politically controlled media through a reduced number of channels and vehicles of communication to the more critical news presented in a liberalized media through a variety of channels and vehicles. Journalists and political experts became the main vehicles to translate political messages for public opinion, having a big potential to influence the politicians’ name recognition, the trust invested in them and voting behavior. In Romania, during the last decade of the communist regime mass media served mainly as an instrument to glorify the personality of the head of state and the communist ideology. The international exchange of cultural and scientific information was banned, the access to books published in developed countries was cut. For this reason, we believe that information consumption should also take into account the activities of reading books from public libraries. During communism, listening to foreign radio stations like Voice of America and Radio Free Europe was considered a political crime, and punished accordingly. In 1985, the regional radio stations were closed down, and the national television programs were reduced to two hours of emission per day. Because of this, after 1989 the free access to information became synonymous with freedom and democracy, and mass media became the main instrument of forming a civil society. Some national news presenters and political talk-show hosts (namely Andreea Esca, Cristian Tudor Popescu and Ion Cristoiu) were so famous, they were included in history manuals. In Brașov County, the psychologist Onelia Pescaru gained popularity as a famous and appreciated journalist, news presenter, and talk-show host. From 2000, she was the editor-in-chef and the director of the local media group MIX (including a TV station, a newspaper and a radio station), and in 2008 she was a candidate for the parliamentary elections. Local press and television broadcast stations appeared in the first decade of post-communism, the most popular in Brașov County being RTT and PROTV Brașov.

In Romania, households access to the Internet was available only in the second decade of the transition. The percentage of households connected to the Internet increased in Romania from 6% in 2004 to 54% in 2012 [62]. In 2012, in a research conducted by EUROSTAT in the Central region of Romania (the region containing the Brasov County), half of the respondents declared that they had never used the Internet [63]. In 2012, in the same region the Internet was used mostly for e-mail (81%), only 19% of Internet users declared that they have downloaded official forms from public authorities’ websites, and only 8% managed to send back the completed forms [64]. According to the European Union statistics [65], in 2012 in Romania political information consumption included mainly television (94%), followed by newspapers (46%) and radio (44%), with the Internet being the least popular option (23%). In Romania, the Internet is not trusted as a viable source of political information and most of the people lack the competencies to use it as such. This skepticism is also reflected in the reticence of the public in providing detailed information about their online activities. Based on this situation, we decided to focus mostly on the measurements of information consumption in terms of exposure to traditional media and to include only one variable regarding Internet consumption (namely, the exposure to news on the Internet).

Another characteristic of post-communist countries is the explosion of international migration in search of job opportunities [3]. In Romania, the rate of international migration evolved from 46.8 migrants per 1000 residents of the population in 2002, to 116.5 in 2012 [66]. National studies have shown that migration has strong effects on individual status and family life, being a source of relational capital, of material resources, and new mentalities and attitudes towards public institutions and political life and politicians [67]. The migration experience creates new social fields of complex transnational networks, interactions, and identities, which overlap the traditional attachment with a certain material space of birth or of residence [68]. Therefore, we decided to use migration experience as an indicator of the socioeconomic status of individuals, assuming that it can be a potential predictor of some indicators of the political dimensions of social capital.

#### Dimensions, indicators, and variables

**The political dimensions of social capital** were measured using 6 variables: voting intention (SC1), voter turnout for local elections (SC2), voter turnout for parliamentary elections (SC3), voter turnout for the National Referendum (SC4), trust in local politicians (SC5) and local politicians’ name recognition (SC6).

For measuring SC1, the following question was asked "If local elections were to take place next Sunday, would you go to vote?", with the following response options: "certainly yes", "probably yes", "probably no", "certainly no", out of which a dummy variable was created, the response option "certainly yes" taking the value 1, and the others the value 0. For the SC2, SC3 and SC4 variables, aggregate data on locality level have been used, such as: locality percentage of actual voter turnout for the 2012 local elections for the president of the County Council and the members of the County Council (SC2); the locality percentage of actual voter turnout for the 2012 parliamentary elections for members of the Senate and the Deputy Chamber (SC2); the locality percentage of actual voter turnout for the 2012 Referendum for the impeachment of the President of Romania (SC3). For creating SC4 and SC4 variables, a 15 item scale (Cronbach's Alpha = .98) was used, which reiterate the question "How much trust do you have in. . .?" for 15 local politicians, with 5 response options: "a great deal", "fair amount", "little", "very little", "I do not know him/her". For measuring SC5, the scale items were recoded into dummy variables where the response options "fair amount of trust" and "a great deal of trust" take the value 1, and “the others” take the value 0 (*Cronbach*'*s Alpha* = .96), and a summative index was computed, ranging from 1 to 15 (representing how many politicians out of a total of 15 are invested with a fair amount of trust and with a great deal of trust). For measuring SC6, the scale items were recoded into dummy variables, where the response option "I do not know him/her" takes the value 0, and “the others” take the value 1 (*Cronbach*'*s Alpha* = .92), and a summative index was computed, ranging from 1 to 15 (representing how many politicians out of a total of 15 are recognized by their name).

**Urbanism** was measured using 15 variables: locality surface area (U1), population density (U2), dwelling density (U3), living density (U4), claiming the need to solve problems associated to the infrastructure of road transport (U5), problems associated to locality modernization (U6), access to public utilities (U7), the management and protection of the environment (U8), mobility and public transport (U9), administrative status as urban locality (U10) and as Municipality (U11), distance from the respondent's house to the centre of Brașov Municipality (U12), locality access to public services (U13) and to public utilities (U14), number of libraries in the locality (U15). The difference between U7 and U14 is that U7 was a subjective measure (the respondent declaring that in the locality in which he lives there is an urgent need to solve problems associated to the access to public utilities), while U14 an objective one (a summative index representing the total number of public utilities units in the locality).

In order to measure U5, U6, U7, U8 and U9 variables, an open question was used "What are the main local problems that should be addressed with priority". The responses were coded into 15 types of social problems, which, by recoding, became 15 dummy variables (using the code 1 for claiming a specific problem and code 0 for claiming the other problems). Among them, only those social problems were selected which fall within the concept of urbanism and are associated with indicators of the political dimensions of social capital. The U12 variable was completed after data collection, starting with the address provided by respondents and calculating, using an online portal (www.distanta.ro), the shortest distance in kilometers that can be covered by car, by bicycle, by public transport or on foot, from the respondents' homes to the Civic Centre of Braşov Municipality. For building the other variables (U1, U2, U3, U4, U10, U11, U13, U14 and U15), aggregate data at locality level were used. U3 was calculated as the ratio between the locality surface area and the dwelling stock of the locality, and U4 as a ratio between the living floor space of the locality and its population. U13 and U14 are summative indices. U13 is the sum of three variables: the total length of drinking water network of the locality, the length of sewerage pipes and of natural gas distribution pipes (*Cronbach*'*s Alpha* = .73); and U14 is the sum of: number of public education units in the locality, the number of public sanitary units and the number of libraries in the locality (*Cronbach*'*s Alpha* = .73).

**Information consumption** was measured using 9 variables: preference for a specific TV talk-show host, namely Onelia Pescaru (IC1), identifying news as the favorite TV show (IC2), exposure to the news on local radio stations (IC3), exposure to the news in local press (IC4), exposure to online news (IC5), exposure to news on TV (IC6), exposure to the news on local stations, RTT and PROTV respectively (IC7), number of information channels on news (IC8), number of active readers at the local library (IC9). For measuring the IC1 variable, the following open question was used: "Who is your favorite TV host". Subsequently, the responses were coded and a dummy variable was created, with the response options 1 for favoring "Onelia Pescaru" and 0 for favoring other TV hosts. Likewise, the IC2 variable was measured by the open question "What is your favorite TV show", creating a dummy variable with the response option 1 for favoring "news" and 0 for favoring other TV shows. For the IC3, IC4, IC5, IC6 variables, respondents were asked, in 4 questions, if they expose themselves or not to news on any of the four information channels, namely radio, printed media, online, TV, and 4 dummy variables were created (value 1 meaning exposure and 0 meaning lack of exposure-). The IC7 variable was measured by the question "On which TV station do you watch most often the local news", and a dummy variable was created, taking value 1 for the response options "RTT" and "PROTV", and value 0 for the other response options. The IC8 variable is a summative index computing 6 dummy variables referring to the presence or absence of exposure to news by means of the following information channels: local newspapers, newspapers distributed at no charge, news websites, local radio stations, central radio stations, television (*Cronbach*'*s Alpha* = .42); the index takes values from 0 to 6, representing the number of media channels by which the respondent is exposed to the news. For building IC9, variable, local aggregated data were used.

**Socioeconomic status** was measured by 8 variables: male gender (SES1), years of age (SES2), level of education, with three response options: primary, secondary, tertiary (SES3), marital status: married (SES4), marital status: not married (SES5), subjective wealth (SES6), minority nationality (SES7), migration experience (SES8), household income (SES9). SES1, SES4, SES5, SES7, and SES8 are dummy variables. The subjective wealth was measured by the question "On a scale from 1 to 10, 1 meaning poor and 10 meaning rich, where do you position yourself?" The migration experience was measured by the question "Is anyone from your family currently working abroad?"; the answer "yes" receiving the code 1 and "no" the code 0.

For a synthetic presentation of the abbreviations of the variables included in the operationalization scheme, see Table A in S1 Appendix.

#### Assumptions

Based on the literature, we assumed that: 1) the distinction between urban and rural is still relevant if the influence of “the rank of the locality” on other dimensions of urbanism and on political dimensions of social capital is statistically significant; 2) the analyzed area is a monocentric one if the negative influence of the “distance from the respondent's house to the center of the core city” on other dimensions of urbanism and on political dimensions of social capital is statistically significant (the analyzed area has urban sprawl issues if as the “distance from the respondent's house to the center of the core city” increases, the locality access to public utilities and to public services decreases); 3) the analyzed area is starting to become a space of flows if most of the indicators of information consumption, some indicators of urbanism (like the presence of the transport infrastructure issue on citizen’s agenda) and some indicators of individual differences (like the presence of migration experience) have a statistically significant influence on the analyzed political dimensions of social capital.

### Statistical analyses

After performing the descriptive statistics, we applied the Hierarchical Regression method, a version of the multiple linear regressions, which enables the input of control variables.

*Multiple Linear Regression* allows expressing the functional relation between a dependent variable and several independent variables by using an equation such as: *Y* = a+*b*_1_*X*_1_ + *b*_2_*X*_2_ +…+ *b*_*n*_*X*_*n*_ which allows determining the regression line, which is the best straight line to express the relation between the analyzed variables (S1 Anexes). In this equation: *a* is the constant (*intercept*), namely the point in which the regression line crosses the Y axis, or, in other words, the predicted value of Y when *X* = 0; *b* is the slope of the regression line which denotes the average quantity of change in Y, as a result of a change in X by one unit. In multiple regressions, the b coefficient does not allow comparisons between the powers of effects of each of the independent variables over the dependent variable, because it is expressed in different measurement units. Such comparisons can be performed by using the standardized regression coefficient (*beta*), which measures the changes in the dependent variable, measured in standard deviations, as the effect of the change in the independent variable by a standard deviation [69]. The regression method can be used to make estimations at the population level, based on the results obtained at the sample level. In this case, the regression equation becomes: $\overset{⌢}{Y}=\alpha +\beta_{1}X_{1}+\beta_{2}X_{2}+\ldots +\beta_{n}X_{n}+\varepsilon$ (where, *β* is the estimated coefficient and *ε* is the error term). *Error term* represents the part of Y variation, which $X_{1},X_{2,},\ldots ,X_{n}$ cannot estimate, assuming that there is always an unpredictable area due to some interactional effects between the independent variables in the model, or due to the variables not included in the model which influences the dependent variable, or due to potential random variations of the dependent variable [70]. The regression method has not only a predictive function but also an explanatory function, which can be evaluated by the *coefficient of determination R*^2^, which denotes the variation of the dependent variable explained by the independent variable [71]. *Adjusted coefficient of determination* is the value of *R*^2^ for which the effect of the number of predictors is eliminated.

*Hierarchical Regression* allows the input of several regression models, which are linked to different coefficients of determination (and thus, different explanatory functions). The differences between these coefficients are tested in terms of statistical significance by the F test. In practice, in order to use the control variables in the hierarchical regression, we proceed as follows: the dependent variable remains the same, and the independent variables are changed from one model to the other. We aim to find a model which brings a statistically significant increase of the explanatory function, meaning the biggest *Adjusted R Square* model is selected, and in this case, if *R Square Change* ≠ 0, *Sig*. *F Change* < .05, then that model brings an improvement to the previous one, and it may replace it [72].

To identify the predictors of the variables that measure the political dimensions of social capital, we built 3 regression models for each of them (SC1, SC2, SC3, SC4, SC5, SC6). For the first model, we used urbanism and information consumption indicators as predictors, and for the second model, we added to the former the indicators of socioeconomic status to analyze, by hierarchical regression, if these indicators induce or not a statistically significant change in the model. The third model is the optimized model, as it was built by introducing only those variables whose coefficients are statistically significant and eliminating those variables that do not meet the conditions of regression application (especially normality, homoscedasticity, lack of multi collinearly, lack of extreme values and influential cases). In particular, we aimed that *Tolerance T* > .2, *Variance* − *Inflation Factor VIF* < 5, and the Dubbin-Watson D to tend to 2.

Overall, we identified 11 statistically significant regression models, including 3 models for examining whether the analyzed area has the characteristics of a mono centric or polycentric spatial structure, 2 models for examining whether the rural-urban distinction is or is not relevant and 6 models to identify predictors of social capital.

## Results

### Descriptive statistics

The results of the descriptive statistics analysis are presented in detail in Table A in S2 Appendix.

#### The characteristics of urbanism, information consumption, and political participation

Within Brașov metropolitan area, in the localities adjacent to Brașov Municipality the declared voting intention at the individual level is very high (85%). Despite the statements of the respondents, the aggregate data at locality level suggest that actual turnout is much smaller than claimed, with an average of 59% in the local elections, 41% in parliamentary elections, and 45% in the national referendum held for the president's impeachment. Furthermore, trust in local politicians and their name recognition are relatively low, from a list of 15 names, people from Brașov declared to know on average only three politicians and trust only one.

According to aggregate data at locality level, the average surface area of the analyzed localities is 155km2, the population density is 113 inhabitants / km2, the dwellings’ density is under one house per km2, each inhabitant being allocated on average a 16m2 of living floor space. On average, 15 public service units operate in each locality (5 of which are libraries) and there is a network of 115km of public utilities (water, sewerage, and natural gas). Individual data showed that the average distance from the respondents' houses to the centre of Brașov Municipality is 17km.

In terms of information consumption, it is interesting that there is only one local TV talk-show host with a high reputation (77% of the respondents declaring they have a favorite TV host, namely Onelia Pescaru, a local TV talk show host). Likewise, the respondents have a high interest in local informative shows (for more than 80% of the respondents declaring they have a favorite TV show, namely the local news show). Most of the respondents (69%) watch TV news, of which 42% specify the local TV stations RTT and PROTV. Around half of the respondents listen to the local news on the radio, and again half of the respondents read the press for local news. However, the exposure to information by electronic means is relatively low (only 8% of the respondents access news online). Aggregate data show that, on average, the number of active readers in libraries is over 2500 in each locality, with an average of around 444 readers in one library.

### Regression Analysis

By applying the simple linear regression method, we identified 5 statistically significant regression models, which predict 3 variables for measuring the concept of urbanism (“the population density of the locality”, “the access to public services”, and “the access to public utilities”), while another 2 variables are measuring the concept of urbanism (“the distance from the respondent’s residence to the centre of Brașov Municipality” and “the urban rank of the locality”) (see Table 2). The estimation accuracy ranges between 1% and 59%, the highest values corresponding to the model that predicts “the access to public utilities” by “the urban rank of the locality”, and the model which predicts “the access to public services” by “the distance from the respondent’s residence to the centre of Brașov Municipality”.

**Table 2:** Regression models using "distance to the center of Brașov municipality" and "population density" as predictors.

Model	DP	IV	Regression equations S*tg*.*F <* .05, S*tg*.*t <* .05	Adj. R square	Beta coefficient
1a	U2	U12	*U*_2_ = 126.410 - .778 *U*_12_	.020	-.148
2a	U13	U12	*U*_13_ = - 28.958 + 2.549 *U*_12_	.446	.669
2b	U13	U10	*U*_13_ = 62130.285 + 22.697 *U*_10_	.189	.436
3a	U14	U12	*U*_14_ = 134.612 - .857 *U*_12_	.009	-.102
3b	U14	U10	U_14_ = 966412.982 + 89.516 *U*_10_	.587	.766

By applying the hierarchical regression method, we identified 6 regression equations, for each of the 6 variables measuring the political dimensions of the social capital (see Table 3). The estimation accuracy ranges between 20% and 82%, with a higher value for the cases where the dependent variables are measured at the aggregate level, in contrast to the cases where these variables are measured at the individual level. The results of the regression analyses are presented, in detail, in Tables A-F in S4 Appendix, and Tables A-G in S5 Appendix.

**Table 3:** The regression of the 6 variables measuring the political dimensions of social capital according to the indicators of urbanism and to the indicators of information consumption.

Model	Regression equations S*tg*.*F <* .05, S*tg*.*t <* .05	Adj. R square
1.3	*SC*_1_ = .812 + .264 *IC*_1_ - .26 *U*_5_	.256
2.3	*SC*_2_ = 53.353 + 97.258 *U*_3_−1.597 *U*_30_ + .23 *U*_12_ - .008 *IC*_9_/*U*_15_ + 1.113 *U*_4_/*U*_1_	.743
3.3	*SC*_3_ = 40.653+184.460 *U*_3_−1.321 *U*_7_ + .032 *U*_13_ - .005 *IC*_9_/*U*_15_ + 1.113 *U*_4_/*U*_1_	.665
4.3	*SC*_4_ = 1.114+0.19 *U*_1_−117.340 *U*_3_+1.889 *U*_4_+1.618 *U*_30_+.56 *U*_12_+.004 *IC*_9_/*U*_15_	.822
5.3	*SC*_5_ = - 3.288 + .261 *U*_12_ + 7.117 *CI*_5_	.364
6.3	*SC*_6_ = 4.668–2.2173 *IC*_2_ + 4.545 *SES*_8_	.200

#### The impact of some specific certain urbanism indicators on other urbanism indicators

In order to ascertain whether the distinction rural-urban is relevant or not within the analyzed area (see Table 2), we built three regression models (model 1b, model 2b, model 3b). The influence of the urban rank of the locality on population density could not be evidenced (model 1b) (see Table B in S4 Appendix). Conversely, the urban rank of the locality significantly influences the access to public services (model 2b) (see Table D in S4 Appendix) and public utilities (model 3b) (see Table F in S4 Appendix). The second model has good predictive power (59%) and suggests that urban localities tend to have access to more public services than rural ones.

In order to ascertain whether the analyzed area has or doesn’t have the features of a monocentric spatial structure (see Table 2), we have highlighted three regression models (model 1a, model 2a, and model 3a). We started from the assumption that a monocentric spatial structure implies that, as the distance between the residences and the core city increases, the population density, the access to public services and access to public utilities decreases. According to the identified models, as the distance to the core city increases, the density of the population decreases (model 1a) (see Table A in S4 Appendix), the access to public utilities decreases (model 3a) (see Table E in S4 Appendix), but the access to public services increases (model 2a) (see Table C in S4 Appendix). Taking into account that model 1a and model 3a, which support the monocentric spatial structure, have low estimation accuracy (20% and 1%), while model 2a, which supports the polycentric structure, has a higher estimation accuracy (45%), we may say that an emerging form of polycentric spatial structure becomes visible in the localities adjacent to Brașov metropolitan region. The model with the best predictive power suggests that people living far from the core city have access to a larger number of public services, compared to people living closer to the core city. A comparison between the much lower (less than 1%) predictive power of the model emphasizing the negative influence of the distance to Brașov Municipality in terms of access to public utilities, and the predictive power of the model stressing the positive influence of the distance to Brașov Municipality regarding access to public services (45%), might raise the issue of the occurrence of the negative effects of urban sprawl phenomenon in the analyzed area.

#### The impact of urbanism indicators on the indicators of the political dimensions of social capital

The variables which measure urbanism with an impact on certain indicators of the political dimensions of the social capital are (see Table 3): locality surface area, dwelling density, living density, perceived need for problem solving associated with the infrastructure of road transport within the locality, administrative status as urban locality, distance from the respondent's house to the center of the core city, number of public services units within the locality and the density of the infrastructure network for access to public utilities. We could not highlight any impact of the following variables, as indicators of urbanism, on any indicators of the political dimensions of social capital: population density, locality's administrative status as Municipality, perceived need to solve problems associated with locality modernization, access to public utilities, environment management and protection, mobility and public transport.

#### The impact of the indicators of information consumption on the indicators of the political dimensions of social capital

The variables measuring information consumption, with an impact on certain indicators of the political dimensions of social capital, are (see Table 3): preference for a specific TV talk-show host (Onelia Pescaru), preference for TV news shows, using the Internet for reading the news and the number of local active readers per library. We could not highlight any impact of the following variables, as indicators of information consumption, on any indicators of the political dimensions of social capital: exposure to news broadcasted on local radio stations, in local press and television, as well as watching news on local TV stations with the highest ratings (namely RTT and PROTV Brașov). Except for the migration experience variable, we could not highlight any influence of the variables measuring socioeconomic status of the respondent (sex, age, education, income, marital status, nationality, perceived welfare) on any indicators of the political dimensions of social capital.

#### The direction and intensity of the influences

The direction and intensity of the influences were identified by analyzing the sign and the value of the standardized (beta) regression coefficients (see Table 4).

**Table 4:** Standardized regression coefficients obtained by regressing the variables measuring the political dimensions of social capital.

Beta	Dependent variables					
Independent variables	SC1	SC2	SC3	SC4	SC5	SC6
U1				.183		
U3		.231	.537	-.107		
U4			-.124	.880		
U5	-.260					
U10		-.277		.069		
U12		.507		.330	.466	
U13			.197			
U14/U1		.187				
IC1	.456					
IC 2						-.241
IC 5					.415	
IC9/U15		-.440	-.283	.092		
SES8						4.542

According to the results, we can predict that more people will declare that they will participate in local elections when their agenda includes no issues related to the infrastructure of road transport of the locality they live in, and when they favor a specific local TV talk-show host, namely Onelia Pescaru (see Table A in S5 Appendix). Also, the percentage of voter turnout for local elections will be larger in: rural localities, localities with a higher dwelling density, localities with a higher density of public utility infrastructure, localities where the dwellings are situated far from the center of Brașov Municipality, localities with a smaller number of active readers per library (see Table B in S5 Appendix). As regards voter turnout for parliamentary elections, the percentage will be higher in: localities with a higher dwelling density, localities with a larger number of public services, rural localities, and localities with a smaller number of active readers per library (see Table C in S5 Appendix). With respect to voter turnout for the referendum, its percentage will be larger in: localities with a larger surface area, localities with a higher living density, urban localities, localities where the dwellings are situated far from the center of Brașov Municipality, localities with a lower dwelling density (see Table D in S5 Appendix). People whose residence is far from the center of Brașov Municipality and people who access news on the Internet will trust a larger number of local politicians (see Table E in S5 Appendix). Similarly, people with migration experience and people who favor TV shows other than news will know a larger number of local politicians (see Table F in S5 Appendix). Therefore, the most common determinants of the indicators of the political dimensions of social capital are the following: the locality’ dwelling density, the distance from the citizen’s house to the center of the core city, and the locality’ number of active readers per library.

Among the variables included in the models (see Table 4), the most significant influence on voting intention occurs when the respondent favors a certain TV talk-show host (namely Onelia Pescaru). The distance from the respondent's residence to the center of Brașov Municipality has the greatest impact on the percentage of voter turnout for local elections. As regards the percentage of voter turnout for parliamentary elections, the greatest impact is associated with the dwelling density and for the case of the percentage of voter turnout for the national referendum, to the living density. The most accurate predictor of trust in local politicians is the distance between the respondent's residence and the center of Brașov Municipality, and of the local politicians’ name recognition, the migration experience.

## Discussion

The aim of this paper is to show how some theoretical models of urban sociology and certain specific features of post-communist countries could be useful in order to study voting behavior. The main objective is to identify what are the characteristics of the spatial structures within the metropolitan area of Brașov and how they influence the political dimensions of social capital. Some dimensions of urbanism, identified as predictors of the political dimensions of social capital, suggest that the area under analysis is predominantly monocentric, where the rural-urban distinction continues to remain relevant. There are also arguments favoring the beginning of the dissolution of the rural-urban distinction and the emergence of polycentric spatial structures and space of flows. We found that the indicators of urbanism, information consumption, and migration experience influence indicators of the political dimensions of social capital. The interpretation of the most powerful predictors of political dimensions of social capital revealed five conclusions. People with migration experience will recognize more local politicians by their name. People living far from the core city will trust more local politicians and will tend to vote in local elections. People living in localities with a denser living floor space will tend to vote in national referendums. People living in localities where dwellings are dense will tend to vote in parliamentary elections. People favoring a certain local talk-show host will tend to declare that they intend to participate in local elections. Overall, our results indicate that, in the study of voting behavior in former communist countries, the conceptualization of urbanism as predictor should consider not only the static material dimensions of space, but also informational space (infrastructure of information consumption and preferences for information consumption), and space as mobility (transport infrastructure, migration experience).

We interpret these results as signs of a complex mechanism wherein not only space structures determine political participation, but also space structures are changing under the influence of global, local, and individual factors. On the one hand, political and economical changes within societies are projected into the spatial structures. At the international level, under the influence of globalization, there is a paradigm shift in urban sociology, from *central place model* (based on urban-rural distinction and on monocentric space structures) [41] to *network society* (based on space of flows and polycentric space structures) [73]. The first model implies the dominance of static and localized space on social action while the second the dominance of global mobility and infrastructure of information consumption. Even so, in post-communist countries like Romania, these transformations take time, creating a situation in which old spatial characteristics coexist with new ones. In this context, some political dimensions of social capital are influenced by the old spatial structures, while others by the emerging ones. On the other hand, through political participation citizens can influence the spatial structure of their localities, by exercising their *right to the city* [74]. The inhabitants of the cities have the right to participate in the public sphere, to decide spatial arrangements meeting their needs to a higher degree. Using social capital and access to information, the citizens could influence in an informed manner the structure of their urban space through the politicians and the parties elected and trusted to represent them.

### What are the characteristics of spatial structures in the analyzed area?

Contrary to the predictions that the world will become urban in the future as stated in literature [7, 34], our study shows that the **distinction rural-urban** continues to be relevant in the analyzed area, given its influence on the access of locality to public utilities and public services. However, there are some results, which might be interpreted as signs of the dissolution of rural-urban distinction. First, the rural or urban administrative status of the locality does not influence the population density. Second, the influence of the locality's urban rank is weaker than the influence of other indicators of urbanism (”the distance between the respondent's residence and the center of Brașov Municipality” or „the living density”) on the indicators of the political dimensions of social capital. Third, the influence of the locality's urban rank is weaker than the influence of the indicators of information consumption (”preference for a specific TV talk-show host” or „exposure to online news”) on the indicators of the political dimensions of social capital. Fourth, the influence of the locality's urban rank is weaker than the influence of migration experience on the indicators of the political dimensions of social capital. More precisely, the classical urban-rural distinction has a weaker influence on voting behavior than other characteristics of material space, of informational space or of individual life experiences.

By demonstrating that, as the residence distance to Brașov Municipality increases, the population density and locality access to public utilities decrease, we reveal that the analyzed area is predominantly **monocentric,** as already highlighted by previous studies [48]. Nevertheless, the finding that the locality's access to public services increases in direct proportion with the residence distance to Brașov Municipality, which has a relatively high estimation accuracy (45%), could represent an argument in favor of the emergence of **polycentric** spatial structures, consistent with the international trends in urban evolution as described in literature [38, 39].

This result is not surprising as there are many other arguments favoring the idea that Brașov metropolitan area could present characteristics of the emergence of a polycentric structure. Despite the fact of being recently created in 2007, Brașov metropolitan area is currently one of the most economically advanced areas throughout Romania. The core city (Brașov Municipality) represents one of the 7 poles of growth determined at national level on the basis on the five criteria: 1) balanced spatial distribution in the territory; 2) location on the cross-national and national transport networks; 3) high level of economic and social development; 4) significant research, development and innovation capacity; 5) historical experience as a regional center with traditional relations with the neighboring cities [75]. In the future, the Integrated Plan for Urban Development envisages connecting Brașov growth pole to the knowledge economy, by investments in Research and Development and in the Information and Communication Technology field in order to increase the international competitiveness of the region. Moreover, since the first half of the last century, one of the biggest universities of the country, Transilvania University, operates in Brașov, currently serving around 20,000 students. In 2012, The Institute of Research, Development, and Innovation: High-Tech Products for a Sustainable Development was established in Brașov, concentrating 27 scientific research centers. Between 1992 and 2012, the population density in Brașov Municipality was reduced to half and the built area increased only by a quarter. Nevertheless, unlike other cities in the country, Brașov Municipality is surrounded by dense enough settlements to lay the foundation for an integrated network of metropolitan transport [76].

One interesting finding is that in Brașov metropolitan area does not present the negative effects of **urban sprawl** phenomenon [49, 50], revealed by recent local studies in the area [52, 53]. This conclusion is sustained by the much lower (under 1%) predictive power of the model emphasizing the negative influence of the residence distance to Brașov Municipality on the access to public utilities. Comparatively, the predictive power of the model emphasizing the positive influence of the residence distance to Brașov Municipality on the access to public services is significantly higher (45%). In other words, the model with the highest predictive power showed that, in the metropolitan area of Brașov, living far from the core city does not imply less access to public services.

The results provide evidence that in the analyzed area the characteristics of the classic distinction between urban and rural and monocentric spatial structures coexist with signs of the emergence of polycentric structures. A possible implication for this might be that in post-communist countries, instead of applying dichotomous models of spatial structures, it is more appropriate to assume the existence of a continuum ranging from rural to urban, and from monocentric to polycentric. Therefore, in metropolitan areas from transitional countries to market economy and political pluralism, we should expect to find the coexistence of certain characteristics of opposing spatial structures.

### How do spatial structures influence the political dimensions of social capital?

It is interesting to note that, in our study, socio-economic status does not influence voting behavior, which contradicts some of the classical theoretical models in the literature [1]. Except for the migration experience, the political dimensions of social capital do not depend on any of the measured individual characteristics of the respondents: gender, age, education, marital status, subjective wealth, nationality, and household income. The interpretation of the direction and intensity of the found influences provides some interesting results. Overall, they suggest four main tendencies. First, people living in rural localities, which have a larger dwellings’ density, and a smaller number of active readers per library tend to participate more in local elections. Second, people living in urban localities, which have a smaller dwellings’ density, but a larger living floor space’ density, a larger surface, and a larger number of active readers per library tend to participate more in national referendums. Third, people living far from the core city, in localities with a denser infrastructure of public utilities, tend to participate more in both local elections and national referendums. Fourth, people connected to global society by reading news on the Internet, and having migration experience, tend to trust and know more local politicians. These tendencies show the complex dynamics between opposing factors like rural-urban, distance-nearness, local-national-global, density-sprawl, and connection-disconnection.

Since most of the predictors of the indicators of the political dimensions of social capital are rather indicators of urbanism than of information consumption, the analyzed area still shows the traditional characteristics of *central place*, stated by Christaller and Lösch in the first half of the last century [41]. The model stipulates the existence of a hierarchy of settlements according to their size and central position, which facilitates the provision of goods and services to the adjacent localities. It entails a hexagonal spatial structure, including a large core city in the center, connected to a hexagonal network of smaller cities, which, in turn, are each connected by hexagonal networks to rural localities. However, the presence of certain influences related to information consumption (preference for a specific TV talk-show host, preference for TV news shows, number of local active readers per library and exposure to Internet news) on the 6 analyzed indicators of the political dimensions of social capital provide evidence for the emergence of the **space of flows** [77, 73]. The emergence of space of flows is also supported by the identified influences of the absence in people’s agendas of issues associated with the transport infrastructure and of migration experience on indicators of political dimensions of social capital. In theory, the space of flows is linked to the development of cross-national hierarchies of cities, which act as strategic nodes on the information market [55]. The new society, called *network society*, not being limited to a geographic space, is structured around networks of electronic processing of information, which facilitate transnational interactions [73]. In our study, the influence of migration experience on the politicians’ name recognition, building on previous studies [67, 68], is another argument favoring the emergence of the transnational space of the *network society* [73]. Therefore, in the metropolitan area of Brașov, the characteristics of material space and its monocentric structure still offers the most of the predictors of voting behavior, but the influence of informational space is also significant and should not be ignored. In the context of globalization and information economy, it is expected that informational space will start to play a stronger role than material space in influencing social actions [54]. We believe that the research of voting behavior in post-communist countries could be furthered by considering both the influence of the characteristics of the local and static dimensions of local spatial structures, and of the emerging space of flows.

The results of the present study bring into the spotlight the impact of urban life on social relations and social actions, summarized in urban sociology by the concept of *urbanism as a way of life*. While classical studies noted the negative influence of population density, locality size and population heterogeneity (measured using indicators of socio-economic status) on social relations [28], this study builds on the previous accomplishments [30–33], confirming some of their conclusions, and infirming others. Unlike the classical measures of urbanism created by Wirth [28], by the present research, we demonstrate that “the population density of the locality” and “the socioeconomic status of the inhabitants” (except for the migration experience) have no impact on the indicators of the political dimensions of social capital. We also demonstrate that “the locality size” influence only one indicator of the political dimension of social capital, namely the voter turnout for the national referendum. However, the urban administrative status of the locality continues to influence some indicators of the political dimensions of social capital (voter turnout for local elections and national referendum). Also, the fact that socioeconomic status, except for the migration experience (which is not a traditional measure of socioeconomic status and which was not used in the classical studies of urbanism), does not significantly influence voting behavior suggests that population heterogeneity is not a relevant dimension of urbanism studied as predictor of voting behavior.

In exploring the effects of urbanism, [28] previous studies showed its negative impact on sociability, [30] its positive impact on community ties, [31] its positive impact on subcultures formation due to the heterogeneity of the population, [32] its negative impact on integration and cohesion, when controlling for the socioeconomic status, and [33] the lack of negative impact on kinship bonds. The present study proves that most of the characteristics of urbanism have a positive impact on voting behavior, apart from the negative influences of dwelling’ density and of living floor space’ density on voter turnout for the national referendum, and of locality's urban status on the voter turnout for local elections. In other words, by studying urbanism as predictor of voting behavior, we bring some arguments against the negative effects of urbanism on social actions and against the classical measure of the concept proposed by Wirth [28] and by Fischer [31]. We believe that of greater importance than highlighting the direction of these influences is focusing on the need of a more complex operationalization of the concept of urbanism, in light of the new evolutions of spatial structures, in order to better understand social capital, in general, and voting behavior, in particular.

Another notable result is that, in the analyzed area, voter turnout is relatively low and so are trust in local politicians and their name’ recognition. These findings are consistent with previous concerns [5, 6, 17] about the dissolution of trust and civic engagement in post-communist countries. An implication of this is the possibility that in post-communist countries low levels of social capital slow down the process of transformation of spatial structures because citizens do not exercise their *right to the city*. This concept was introduced by Lefebvre [78], and developed by Castells [77] and Harvey [79], meaning "the right to information, the right to use of multiple services, the right of users to make known their ideas on the space and time of their activities in urban areas" [74]. It refers not only to having access to resources but also the right to change the spatial structure of a locality according to its inhabitants’ needs [79]. Firstly, these attitudes and behaviors are manifestations of social capital, as defined by Putnam [12]. Secondly, within the context of globalization and information revolution, the exercise of the right to the city depends on the access to informational technologies, especially the Internet [73]. Therefore, social capital and information consumption are means to exercise the right to the city, to change its spatial structure. Overall, in support of a sustainable urban development, through the article we advocate for the idea that more advanced studies are needed, especially in the former communist countries, analyzing emerging forms of space of flows, by focusing on the complex relationships between the dimensions of urbanism, information consumption, and social capital.

### Summary of limitations of the study

The concept of social capital is much more complex than its political dimensions (trust in politicians, politicians’ name recognition, and voter turnout) and information consumption does not reduce to exposure to news and library books. We explained these choices in detail in the Measurements section of the paper. The main limitation of our research is posed by the difficulty of finding and collecting sufficient accurate data in post-communists countries like Romania regarding membership in associations, participation in social protests, informal structures of support and norms of reciprocity, and the private use of electronic communication technologies. During the communist period, such behaviors were discouraged and severely punished, in order to create an atomized society. The overwhelming surveillance performed by the state apparatus structures of the secret police established a climate of fear and suspicion, everyone being regarded as a potential secret agent of the state, and institutionalized duplicitous behavior [80]. In other words, there was a rupture between formal and informal opinions and actions, people developing coping mechanisms based on informal support networks of family and close friends, hidden from the eye of the totalitarian state. These mental schemes survived the communist era [81] and were even reinforced by the post-communist quest and public stigmatization of former collaborators with the secret police and former members of the Communist Party. Even so, it is beyond the scope of our study to examine these effects. It is known from literature that the communism inheritance had negative effects on social capital [5, 6], but further studies need to be conducted regarding the particularities of these effects.

Due to insufficient data, no clear pattern emerged of how spatial structures influence the political dimensions of social capital. The results of the regressions provided evidence that only some dimensions of urbanism and information consumptions can be used to predict the political aspects of social capital measured by this study. In addition, the conclusions about the characteristics of the spatial structures in the analyzed area should be interpreted with caution. It is clear that this geographic area is in a state of flux with respect to spatial development and adoption of information technology; however, more studies are needed to assert a definite move towards a space of flows or a profound change to a polycentric structure.

### Summary of implications for future research

Based on the results of the regression analyses, we recommend that the conceptualization of urbanism as predictor of voting behavior should include additional indicators such as: dwelling density, living density, access to road transport infrastructure, to public utilities and services, and the distance to the center of the city/core cities. Previously, we pointed out that certain indicators of information consumption (especially accessing online news) leave their mark on some indicators of the political dimensions of social capital (especially on trust invested in local politicians), and that the migration experience influences the politicians’ name recognition. Therefore, considering the theorizations of space of flows developed by Castells [73, 77], we expect that the operational measure of urbanism should not ignore the indicators of information consumption and migration experience.

Considering the limits of our study, we advocate for developing in the future more complex and subtle measures of the multiple dimensions of social capital in post-communist countries, like those performed by the Australian Bureau of Statistics [16]. In addition, more data that are accurate need to be collected regarding the specificity of the use of electronic networks of information in countries in transition to the information economy. The access to this type of data will allow for a more complex mathematical analysis of the effects of the emergence of the space of flows on social capital. For instance, further studies might focus on the interactional effects of spatial and informational complex networks on evolutionary cooperation behaviors, using recent evolutionary game theory space models like the spatial prisoner’s dilemma game [82], the evolutionary model of public goods game [83] or the traveler’s dilemma game [84]. These studies could place in a broader context the manner in which the right to the city is exercised in post-communist countries, focusing not only on voting behavior but also on other dimensions of social capital, like evolutionary cooperation behaviors.

## Supporting Information

S1 Anexes (DOC)

S1 Appendix Table A. Explanation of the abbreviations of the variables included in the study. (TIF)

S2 Appendix Table A. Descriptive Statistics. (TIF)

S3 Appendix Table A. Bivariate correlations between the dependent variables and independent variables. (TIF)

S4 Appendix (PDF)

S5 Appendix (PDF)

S1 Aprouval (DOC)

S1 Survey (DOC)
