# Supplementary material for: The Influence of Urbanism and Information Consumption on Political Dimensions of Social Capital: Exploratory Study of the Localities Adjacent to the Core City from Brașov Metropolitan Area, Romania
Source: PLoS One. 2016 Jan 25;11(1):e0144485. doi: 10.1371/journal.pone.0144485 (PMC4726559; doi:10.1371/journal.pone.0144485)
Supplement: S1 Anexes — (DOC) [file pone.0144485.s001.doc]

anexes PLOS ONE_Repanovici.doc

APPENDIX

Table A.1 Explanation of the abbreviations of the variables included in the study

| **Code** | **Variable name** |
| --- | --- |
| **SC** | Social capital |
| **SC1** | voting intention: 1- certainly yes; 0 probably yes |
| **SC2** | percentage of voter turnout to the 2012 local elections, per locality |
| **SC3** | percentage of voter turnout to the 2012 parliamentary elections, per locality |
| **SC4** | percentage of voter turnout to the 2012 presidential impeachment referendum, per locality |
| **SC5** | number of local trustworthy politicians: value ranges from 1 to 15 |
| **SC6** | number of local politicians they know: value ranges from 1 to 15 |
| **U** | Urbanism |
| **U1** | locality surface area in square km |
| **U2** | population density: the ratio between the locality's surface area and its population |
| **U3** | dwelling density: the ratio between the locality surface area and its dwelling stock |
| **U4** | living density: living sqm per inhabitant |
| **U5** | local social problems related to road transport infrastructure: 1- yes; 0- no |
| **U6** | local social problems related to the modernization and landscaping of the locality: 1- yes; 0- no |
| **U7** | local social problems related to the access to public utilities: 1- yes; 0- no |
| **U8** | local social problems related to the management and protection of the environment: 1- yes; 0- no |
| **U9** | county social problems related to mobility and public transport infrastructure: 1- yes; 0- no |
| **U10** | urban locality: 1- yes; 0- no |
| **U11** | municipality: 1- yes; 0- no |
| **U12** | distance measured in km from the respondent's house to the centre of Brașov municipality |
| **U13** | index of access to public services: the sum of healthcare, educational units and libraries |
| **U14** | index of access to utilities: the sum of total length of water, sewer and natural gas networks |
| **U15** | no. of libraries in the locality |
| **U14/U1** | density of local public utility network in km |
| **IC** | Information consumption |
| **IC1** | favourite TV producer Onelia Pescaru: 1- yes; 0- no |
| **IC2** | favourite TV show news: 1- yes; 0- no |
| **IC3** | listens to the news on the local radio stations: 1- yes; 0- no |
| **IC4** | reads the news in the local newspapers: 1- yes; 0- no |
| **IC5** | reads the news online: 1- yes; 0- no |
| **IC6** | watches the news on TV: 1- yes; 0- no |
| **IC7** | watches the news on the local RTT and PROTV stations: 1- yes; 0- no |
| **IC8** | number of information channels on news: value ranges from 0 to 6 |
| **IC9** | number of active library readers |
| **IC9/U15** | number of active library readers per locality |
| **SES** | Socio-economic status (individual) |
| **SES1** | male: 1- yes; 0- no |
| **SES2** | years of age |
| **SES3** | education: primary, secondary, tertiary |
| **SES4** | marital status married: 1- yes; 0- no |
| **SES5** | marital status single: 1- yes; 0- no |
| **SES6** | self-positioning on the poor-rich axis (1-poor; 10-reach) |
| **SES7** | minority nationality: 1- yes; 0- no |
| **SES8** | migration experience: 1- yes; 0- no |
| **SES9** | income per household in RON (Romanian national currency) |

Table A. 2 Descriptive Statistics

| **Abbr.** | **Type of data collection** | **Unit** | **N** | **Minimum** | **Maximum** | **Mean/Proportion** |
| --- | --- | --- | --- | --- | --- | --- |
| **SC** |  |  |  |  |  |  |
| **SC1** | Individual | Presence of the characteristic | 600 | 0 | 1 | 84.7% |
| **SC2** | Aggregate | % inhabitants in the locality | 600 | 53.33 | 74.18 | 58.66 |
| **SC3** | Aggregate | % inhabitants in the locality | 600 | 34.87 | 55.86 | 40.80 |
| **SC4** | Aggregate | % inhabitants in the locality | 600 | 17.09 | 92.15 | 45.29 |
| **SC5** | Individual | Local politicians | 37 | 0 | 15 | 0.89 |
| **SC6** | Individual | Local politicians | 548 | 0 | 15 | 3.42 |
| **U** |  |  |  |  |  |  |
| **U1** | Aggregate | square km | 600 | 27.73 | 320 | 155.05 |
| **U2** | Aggregate | Inhabitant/square km | 600 | 47 | 167 | 113.12 |
| **U3** | Aggregate | House/square km | 600 | 0.1 | 0.8 | 0.27 |
| **U4** | Aggregate | Living sqm/inhabitant | 600 | 10 | 39 | 16.34 |
| **U5** | Individual | Presence of the characteristic | 564 | 0 | 1 | 17.9% |
| **U6** | Individual | Presence of the characteristic | 564 | 0 | 1 | 1.6% |
| **U7** | Individual | Presence of the characteristic | 564 | 0 | 1 | 15.6% |
| **U8** | Individual | Presence of the characteristic | 564 | 0 | 1 | 5.1% |
| **U9** | Individual | Presence/absence of the characteristic | 564 | 0 | 1 | 5.5% |
| **U10** | Aggregate | Presence of the characteristic | 564 | 0 | 1 | 69.3% |
| **U11** | Aggregate | Presence of the characteristic | 600 | 0 | 1 | 34.2% |
| **U12** | Individual | km | 600 | 7 | 31 | 17.2 |
| **U13** | Aggregate | Public service units | 600 | 0 | 60 | 14.87 |
| **U14** | Aggregate | km of utility infrastructure | 600 | 29 | 201.6 | 119.87 |
| **U15** | Aggregate | libraries | 600 | 1 | 10 | 5.14 |
| **U14/U1** | Aggregate | km of networks/square km | 600 | 0.21 | 7.27 | 1.02 |
| **IC** |  |  |  |  |  |  |
| **IC1** | Individual | Presence of the characteristic | 48 | 0 | 1 | 77.1% |
| **IC2** | Individual | Presence of the characteristic | 118 | 0 | 1 | 80.5% |
| **IC3** | Individual | Presence of the characteristic | 600 | 0 | 1 | 53.2% |
| **IC4** | Individual | Presence of the characteristic | 600 | 0 | 1 | 50% |
| **IC5** | Individual | Presence of the characteristic | 600 | 0 | 1 | 7.7% |
| **IC6** | Individual | Presence of the characteristic | 595 | 0 | 1 | 67.9% |
| **IC7** | Individual | Presence of the characteristic | 404 | 0 | 1 | 42.1% |
| **IC8** | Individual | Information channels | 595 | 0 | 6 | 2.58 |
| **IC9** | Aggregate | Readers | 600 | 97 | 5515 | 2589.21 |
| **IC9/U15** | Aggregate | Active readers/library | 600 | 76 | 881 | 443.77 |
| **SES** |  |  |  |  |  |  |
| **SES1** | Individual | Presence of the characteristic | 600 | 0 | 1 | 49.3% |
| **SES2** | Individual | years | 599 | 18 | 89 | 50 |
| **SES3** | Individual | Education level | 597 | 1 | 3 | 1.72 |
| **SES4** | Individual | Presence of the characteristic | 599 | 0 | 1 | 61.6% |
| **SES5** | Individual | Presence of the characteristic | 599 | 0 | 1 | 19.5% |
| **SES6** | Individual | Level of self-perceived welfare | 587 | 1 | 10 | 4.42 |
| **SES7** | Individual | Presence of the characteristic | 597 | 0 | 1 | 11.4% |
| **SES8** | Individual | Presence of the characteristic | 598 | 0 | 1 | 9.5% |
| **SES9** | Individual | RON | 490 | 0 | 10000 | 1535.96 |

Table A.3 Bivariate correlations between the dependent variables and independent variables

| **Pearson Correlations Bivariate** | **Dependent variables** | | | | | |
| --- | --- | --- | --- | --- | --- | --- |
| **Independent variables:** | SC1 | SC2 | SC3 | SC4 | SC5 | SC6 |
| **Urbanism** |  |  |  |  |  |  |
| **U1** | -.153* | -.282* |  | -.169* |  |  |
| **U2** |  | -.394* | -.594* |  |  | .138* |
| **U3** |  | .569* | .721* | -.371* |  | -.100* |
| **U4** |  |  | -.100* | .796* |  |  |
| **U5** | -.113* |  | .117* |  |  | -179* |
| **U6** |  |  |  | .200* |  |  |
| **U7** |  |  | -.119* |  |  |  |
| **U8** |  |  |  | .118* |  |  |
| **U9** |  | -.120* |  |  |  |  |
| **U10** |  | -.443* |  | .185* |  |  |
| **U11** | -.103* | -.565* | -.345* | -.268* |  | .197* |
| **U12** |  | .302* | .199* | .308* | .476* | -.127* |
| **U13** |  |  | -.127* |  | .408* |  |
| **U14** |  | -.331* |  |  |  |  |
| **U15** |  | -.419* |  | -.274* |  |  |
| **U14/U1** |  |  |  | .411* |  |  |
| **Information Consumption** |  |  |  |  |  |  |
| **IC1** | .374* |  |  |  |  |  |
| **IC2** |  | .262* | .202* |  |  | -.256* |
| **IC3** | .129* |  |  |  |  |  |
| **IC4** |  | -.103* |  |  |  | .103* |
| **IC5** |  |  |  |  | .426* |  |
| **IC6** |  | -.173* | -.129* |  |  | .158* |
| **IC7** |  | .104* |  |  | -.352* |  |
| **IC8** |  | -.162* |  |  |  | .133* |
| **IC9** |  | -.623* | -.304* | -.251* |  | .130* |
| **IC9/U15** |  | -.720* | -.512* |  | -.197* |  |
| **Socio-Economic Status** |  |  |  |  |  |  |
| **SES1** |  |  |  |  |  | .101* |
| **SES2** |  | -.104* |  |  |  | .110* |
| **SES3** |  | -.133* |  |  |  |  |
| **SES4** |  | -.118* |  |  |  | .169* |
| **SES5** |  | .140* | .146* |  |  | -.123* |
| **SES6** |  | -.146* |  |  |  |  |
| **SES7** |  | .106* | .146* |  |  |  |
| **SES8** |  |  |  |  |  | .192* |
| **SES9** |  |  |  |  |  | .105* |

*The table records only statistically significant $r>.1$values ($Sig.<.05$)

Table A.4 Regression of the ”population density” variable according to the ”distance to the downtown area of Brașov” variable

| **DV: U2** | **Model 1a** | | | | |
| --- | --- | --- | --- | --- | --- |
| **IV:** | $F=13.217\text{ }(N=593\text{ }Sig.\mathrm{F}\text{ }=.000<.05)Adjusted\text{ }R\text{ }Square=.200\text{ }Constant=126.411$ | | | | |
|  | $Durbin-Watson\text{ }=\text{ }1.8$ | | | *Collinearity Statistics* | |
| **Urbanism** | B | Beta | t | T | VIF |
| **U12** | -.778* | -.148 | -3.635 | 1 | 1 |

$$DV=dependent\mathrm{variable},$$

$$IV=independent\mathrm{variable}$$

*The table records only the results of the regression analysis for predictors with statistically significant values of B coefficients $(Sig.\text{ }t<.05)$

Table A.5 Regression of the ”population density” variable according to the ”urban administrative status of the locality” variable

| **DV: U2** | **Model 1b** | | | | |
| --- | --- | --- | --- | --- | --- |
| **IV:** | $F=.426\text{ }(N=567\text{ }Sig.\mathrm{F}\text{ }=.514>.05)Adjusted\text{ }R\text{ }Square=-.001\text{ }Constant=408.958$ | | | | |
|  | $Durbin-Watson\text{ }=\text{ }1.9$ | | | *Collinearity Statistics* | |
| **Urbanism** | B | Beta | t | T | VIF |
| **U10** |  |  |  |  |  |

$$DV=dependent\mathrm{variable},$$

$$IV=independent\mathrm{variable}$$

*The table records only the results of the regression analysis for the predictors with statistically significant values of B coefficients $(Sig.\text{ }t<.05)$, The table for model 1b does not comprise information, because the model is not statistically significant $Sig.\mathrm{F}>.05$

Table A.6 Regression of the ”index of access to public services” variable according to the ”distance to the downtown area of Brașov” variable

| **DP: U13** | **Model 2a** | | | | |
| --- | --- | --- | --- | --- | --- |
| **IV:** | $F=8.759\text{ }(N=593\text{ }Sig.\text{ F}=.000<.05)Adjusted\text{ }R\text{ }Square=.446\text{ }Constant=-28.958$ | | | | |
|  | $Durbin-Watson\text{ }=\text{ }1.6$ | | | *Collinearity Statistics* | |
| **Urbanism** | B | Beta | t | T | VIF |
| **U12** | 2.549* | .669 | 21.871 | 1 | 1 |

$$DV=dependent\mathrm{variable},$$

$$IV=independent\mathrm{variable}$$

*The table records only the results of the regression analysis for predictors with statistically significant values of B coefficients $(Sig.\text{ }t<.05)$

Table A.7 Regression of the ”index of access to public services” variable according to the ”urban administrative status of the locality” variable

| **DV: U13** | **Model 2b** | | | | |
| --- | --- | --- | --- | --- | --- |
| **IV:** | $F=132.596\text{ }(N=567\text{ }Sig.\text{ F }=.000<.05)Adjusted\text{ }R\text{ }Square=-.189\text{ }Constant=62130.285$ | | | | |
|  | $Durbin-Watson\text{ }=\text{ }1.3$ | | | *Collinearity Statistics* | |
| **Urbanism** | B | Beta | t | T | VIF |
| **U10** | 22.697* | .436 | 11.505 | 1 | 1 |

$$DV=dependent\mathrm{variable},$$

$$IV=independent\mathrm{variable}$$

*The table records only the results of the regression analysis for predictors with statistically significant values of B coefficients $(Sig.\text{ }t<.05)$

Table A.8 Regression of the ”index of access to public utilities” variable according to the ”distance to the downtown area of Brașov” variable

| **DV: U14** | **Model 3a** | | | | |
| --- | --- | --- | --- | --- | --- |
| **IV:** | $F=6.164\text{ }(N=593\text{ }Sig.\text{ F }=.013<.05)Adjusted\text{ }R\text{ }Square=.009\text{ }Constant=134.612$ | | | | |
|  | $\begin{matrix} Durbin-Watson\text{ }=\text{ }1.2 \end{matrix}$ | | | *Collinearity Statistics* | |
| **Urbanism** | B | Beta | t | T | VIF |
| **U12** | -.857* | -.102 | -2.483 | 1 | 1 |

$$DV=dependent\mathrm{variable},$$

$$IV=independent\mathrm{variable}$$

*The table records only the results of the regression analysis for predictors with statistically significant values of B coefficients $(Sig.\text{ }t<.05)$

Table A.9 Regression of the ”index of access to public utilities” variable according to the”urban administrative status of the locality” variable

| **DV: U14** | **Model 3b** | | | | |
| --- | --- | --- | --- | --- | --- |
| **IV:** | $F=804.323\text{ }(N=567\text{ }Sig.\text{ F }=.000<.05)Adjusted\text{ }R\text{ }Square=-.587\text{ }Constant=966412.982$ | | | | |
|  | $Durbin-Watson\text{ }=\text{ }1.9$ | | | Collinearity Statistics | |
| **Urbanism** | B | Beta | t | T | VIF |
| **U10** | 89.516* | .766 | 28.361 | 1 | 1 |

$$DV=dependent\mathrm{variable},$$

$$IV=independent\mathrm{variable}$$

*The table records only the results of the regression analysis for predictors with statistically significant values of B coefficients $(Sig.\text{ }t<.05)$

Table A.10 Regression of the ”voting intention” variable

| **DV: SC1** | **Model 1.1** | | | **Model 1.2** | | | **Model 1.3** | | | | | |
| --- | --- | --- | --- | --- | --- | --- | --- | --- | --- | --- | --- | --- |
| **IVs:** | $F=7.858\text{ }(Sig.\text{ F }=.000<0.05)$ $Adjusted\text{ }R\text{ }Square=.340$ $Constant=.684$ | | | $F=4.147\text{ }(Sig.\text{ F}=.003<0.05)$ $Adjusted\text{ }R\text{ }Square=.321$ $Constant=.639$ | | | $F=8.759\text{ }(N=47\text{ }Sig.\mathrm{F}\text{ }=.001<.05)$ $Adjusted\text{ }R\text{ }Square=.256$ $Constant=.812$ | | | | | |
|  | $R\text{ }Square\text{ }Change=\text{ }.033\text{ }(Sig.\text{ }F\text{ }Change=.585>.05)$ | | | | | | $Durbin-Watson\text{ }=\text{ }1.8$ | | | *Collinearity Statistics* | | |
| **Urbanism** | B | Beta | t | B | Beta | t | B | Beta | t | T | | VIF |
| **U11** |  |  |  |  |  |  |  |  |  |  | |  |
| **U5** |  |  |  | -.288* | -.288 | -2.067 | -.260* | -.260 | -2.021 | 1 | | 1 |
| **Information Consumption** |  |  |  |  |  |  |  |  |  |  | |  |
| **IC1** | .264* | .544 | 8.048 | .382* | .581 | 4.274 | .264* | .456 | 3.541 | | 1 | 1 |
| **Socio-Economic Status** |  |  |  |  |  |  |  |  |  |  | |  |
| **SES2** |  |  |  |  |  |  |  |  |  | |  |  |
| **SES3** |  |  |  |  |  |  |  |  |  | |  |  |
| **SES9** |  |  |  |  |  |  |  |  |  | |  |  |

$$DV=dependent\mathrm{variable},$$

$$IVs=independent\mathrm{variables}$$

*The table records only the results of the regression analysis for predictors with statistically significant values of B coefficients $(Sig.\text{ }t<.05)$

Table A.11 Regression of the "percentage of voter turnout to the 2012 local elections, per locality" variable

| **DV: SC2** | **Model 2.1** | | | **Model 2.2** | | | **Model 2.3** | | | | |
| --- | --- | --- | --- | --- | --- | --- | --- | --- | --- | --- | --- |
| **IVs:** | $F=86.018\text{ }$ $(Sig.\mathrm{F}\text{ }=.000<.05)$ $Adjusted\text{ }R\text{ }Square=.913$ $Constant=48.835$ | | | $F=56.074\text{ }$ $(Sig.\mathrm{F}\text{ }=.000<.05)$ $Adjusted\text{ }R\text{ }Square=.913$ $Constant=48.312$ | | | $F=53.353$ $(N=560\text{ }Sig.\text{ F}=.000<.05)$ $Adjusted\text{ }R\text{ }Square=.743$ $Constant=53.353$ | | | | |
|  | $R\text{ }Square\text{ }Change=\text{ }.006\text{ }$ $(F\text{ }Change=.959;\text{ }Sig.\text{ }F\text{ }Change=.466>.05)$ | | | | | | $Durbin-Watson\text{ }=\text{ }1.4$ | | | *Collinearity Statistics* | |
| **Urbanism** | B | Beta | t | B | Beta | t | B | Beta | t | T | VIF |
| **U1** |  |  |  |  |  |  |  |  |  |  |  |
| **U2** |  |  |  |  |  |  |  |  |  |  |  |
| **U3** | 95.678* | .204 | 4.208 | .009* | .067 | .892 | 97.258* | .231 | 9.074 | 07 | 14 |
| **U5** |  |  |  |  |  |  |  |  |  |  |  |
| **U10** |  |  |  |  |  |  | -1.597* | -.177 | -6.277 | 06 | 17 |
| **U11** | 19.990* | 2.666 | 13.503 | 18.918* | 2.523 | 11.780 |  |  |  |  |  |
| **U12** | .509* | .908 | 19.232 | .503* | .989 | 17.630 | .332 * | .507 | 21.869 | 08 | 12 |
| **U14** | -.035* | -.503 | -3.449 | -.035* | -.504 | -3.379 |  |  |  |  |  |
| **U15** | 3.402* | 2.715 | 15.072 | 3.233* | 2.580 | 13.235 |  |  |  |  |  |
| **U14/U1** |  |  |  |  |  |  | 1.113* | .187 | 6.990 | 06 | 16 |
| **Information consumption** |  |  |  |  |  |  |  |  |  |  |  |
| **CI2** |  |  |  |  |  |  |  |  |  |  |  |
| **CI7** |  |  |  |  |  |  |  |  |  |  |  |
| **CI8** |  |  |  |  |  |  |  |  |  |  |  |
| **CI9** | -.009* | -4.704 | -15.989 | -.009* | -4.464 | -13.963 |  |  |  |  |  |
| **CI9/U15** |  |  |  |  |  |  | -.008* | -.440 | -14.833 | 05 | 19 |
| **Socio-economic status** |  |  |  |  |  |  |  |  |  |  |  |
| **SES2** |  |  |  |  |  |  |  |  |  |  |  |
| **SES3** |  |  |  | .480* | .078 | 2.113 |  |  |  |  |  |
| **SES4** |  |  |  |  |  |  |  |  |  |  |  |
| **SES5** |  |  |  |  |  |  |  |  |  |  |  |
| **SES6** |  |  |  |  |  |  |  |  |  |  |  |
| **SES7** |  |  |  |  |  |  |  |  |  |  |  |
| **SES8** |  |  |  | -.360* | .-024 | -.773 |  |  |  |  |  |

$$DV=dependent\mathrm{variable},$$

$$IVs=independent\mathrm{variables}$$

*The table records only the results of the regression analysis for predictors with statistically significant values of B coefficients $(Sig.\text{ }t<.05)$

Table A.12 Regression of the "percentage of voter turnout to the 2012 parliamentary elections, per locality" variable

| **DV: SC3** | **Model 3.1** | | | | **Model 3.2** | | | **Model 3.3** | | | | |
| --- | --- | --- | --- | --- | --- | --- | --- | --- | --- | --- | --- | --- |
| **IVs:** | $F=29.841$ $(Sig.\text{ F}=.000<.05)$ $Adjusted\text{ }R\text{ }Square=.702$ $Constant=39.329$ | | | | $F=23.141$ $(Sig.\mathrm{F}\text{ }=.000<.05)$ $Adjusted\text{ }R\text{ }Square=.707$ $Constant=36.241$ | | | $F=245.581$ $(N=564\text{ }Sig.\text{ }=.000<.05)$ $Adjusted\text{ }R\text{ }Square=.665$ $Constant=40.653$ | | | | |
|  | $R\text{ }Square\text{ }Change=\text{ }.012$ $(F\text{ }Change=1.557;\text{ }Sig.\text{ }F\text{ }Change=.205>.05)$ | | | | | | | $Durbin-Watson\text{ }=\text{ }1.5$ | | | *Collinearity Statistics* | |
| **Urbanism** | B | Beta | | t | B | Beta | t | B | Beta | t | T | VIF |
| **U2** | -.044* | -.375 | | -3.382 | -.038* | -.324 | -2.859 |  |  |  |  |  |
| **U3** | 206.327* | .583 | | 7.261 | 234.309* | .662 | 7.020 | 18146* | .537 | 15.406 | 05 | 22 |
| **U4** |  |  | |  |  |  |  |  |  |  |  |  |
| **U7** | -1.530* | -.147 | | -2.684 | -1.319* | -.127 | -2.259 | -1.321* | -.124 | -5.193 | 1 | 1 |
| **U11** | -2.667* | -.395 | | -1.929 | -3.676* | -.544 | -2.504 |  |  |  |  |  |
| **U12** |  |  | |  |  |  |  |  |  |  |  |  |
| **U13** | .041* | .315 | | 2.185 | .051* | .388 | 2.552 | .032* | .197 | 7.336 | 08 | 13 |
| **Information Consumption** |  |  | |  |  |  |  |  |  |  |  |  |
| **CI2** |  |  | |  |  |  |  |  |  |  |  |  |
| **CI9** | .001* | .383 | | 1.903 | .001* | .533 | 2.461 |  |  |  |  |  |
| **CI9/U15** |  |  | |  |  |  |  | -.005* | -.283 | -10.365 | 07 | 13 |
| **Socio-Economic Status** |  |  | |  |  |  |  |  |  |  |  |  |
| **SES5** |  | |  |  |  |  |  |  |  |  |  |  |
| **SES7** |  | |  |  |  |  |  |  |  |  |  |  |

$$DV=dependent\mathrm{variable},$$

$$IVs=independent\mathrm{variables}$$

*The table records only the results of the regression analysis for predictors with statistically significant values of B coefficients $(Sig.\text{ }t<.05)$

Table A.13 Regression of the "percentage of voter turnout to the 2012 presidential impeachment referendum, per locality" variable

| **DV: SC4** | **Model 4.1** | | | **Model 4.2** | | | **Model 4.3** | | | | | | | |
| --- | --- | --- | --- | --- | --- | --- | --- | --- | --- | --- | --- | --- | --- | --- |
| **IVs:** | $F=258.158$ $(Sig.\text{ F}=.000<.05)$ $Adjusted\text{ }R\text{ }Square=.858$ $Constant=9.230$ | | | $F=199.142$ $(Sig.\text{ F}=.000<.05)$ $Adjusted\text{ }R\text{ }Square=.858$ $Constant=9.370$ | | | $F=430.266$ $(N=560,\text{ }Sig.\text{ F}=.000<.05)$ $Adjusted\text{ }R\text{ }Square=.822$ $Constant=1.414$ | | | | | | | |
|  | $R\text{ }Square\text{ }Change=\text{ }.001$ $(F\text{ }Change=1.197;\text{ }Sig.\text{ }F\text{ }Change=.310>.05)$ | | | | | | $Durbin-Watson\text{ }=\text{ }1.5$ | | | | | *Collinearity Statistics* | | |
| **Urbanism** | B | Beta | t | B | Beta | t | B | | Beta | t | | T | VIF | |
| **U1** | .039* | .355 | 9.055 | .039* | .355 | 9.037 | .019* | | .183 | 6.324 | | 04 | 26 | |
| **U3** | -122.018* | -.106 | -4.816 | -120.267* | -.104 | -4.722 | -117.340* | | -.107 | -4.948 | | 07 | 15 | |
| **U4** | 1.765* | .820* | 32.642 | 1.768* | .821 | 32.660 | 1.889* | | .880 | 39.740 | | 06 | 16 | |
| **U6** |  |  |  |  |  |  |  | |  |  | |  |  | |
| **U8** |  |  |  |  |  |  |  | |  |  | |  |  | |
| **U10** | 4.851* | .196 | 5.587 | 4.850* | .196 | 5.523 | 1.618* | | .069 | 2.225 | | 03 | 3 | |
| **U11** | -7.209* | -.318 | -3.221 | -6.999* | -.309 | -3.121 |  | |  |  | |  |  | |
| **U12** | .346* | .202 | 7.184 | .341* | .199 | 6.935 | .560* | | .330 | 16.778 | | 08 | 12 | |
| **U15** | -2.013* | -.538 | -6.275 | -2.029* | -.542 | -6.313 |  | |  |  | |  |  | |
| **Information Consumption** |  |  |  |  |  |  |  | |  | |  |  |  | |
| **CI9/U15** |  |  |  |  |  |  | .004* | | .092 | | 3.850 | 05 | | 18 |
| **CI9** | .003* | .507 | 3.145 | .003* | .503 | 3.117 |  | |  | |  |  | |  |
| **Socio-Economic Status** |  |  |  |  |  |  |  | |  | |  |  | |  |
| **SES2** |  |  |  |  |  |  |  |  | | |  |  | |  |
| **SES3** |  |  |  |  |  |  |  |  | | |  |  | |  |
| **SES9** |  |  |  |  |  |  |  |  | | |  |  | |  |

$$DV=dependent\mathrm{variable},$$

$$IVs=independent\mathrm{variables}$$

*The table records only the results of the regression analysis for predictors with statistically significant values of B coefficients $(Sig.\text{ }t<.05)$

Table A.14 Regression of the ”trust in local politicians” variable

| **DV: SC5** | **Model 1** | | | **Model 2** | | | **Model 3** | | | | |
| --- | --- | --- | --- | --- | --- | --- | --- | --- | --- | --- | --- |
| **IVs:** | $F=7.733$ $(Sig.\text{ F}=.001<.05)$ $Adjusted\text{ }R\text{ }Square=.402$ $Constant=-3.873$ | | | $F=4.113$ $(Sig.\text{ F}=.006<.05)$ $Adjusted\text{ }R\text{ }Square=.384$ $Constant=-6.832$ | | | $F=11.293$ $(N=37\text{ }Sig.\mathrm{F}\text{ }=.000<.05)$ $Adjusted\text{ }R\text{ }Square=.364$ $Constant=-3.288$ | | | | |
|  | $R\text{ }Square\text{ }Change=\text{ }.045$ $(F\text{ }Change=.728;\text{ }Sig.\text{ }F\text{ }Change=.546>.05)$ | | | | | | $Durbin-Watson\text{ }=\text{ }2.6$ | | | *Collinearity Statistics* | |
| **Urbanism** | B | Beta | t | B | Beta | t | B | Beta | t | T | VIF |
| **U12** | .309* | .510 | 2.814 | .313 | .516* | 2.389 | .261* | .466 | 3.507 | 1 | 1 |
| **U13** |  |  |  |  |  |  |  |  |  |  |  |
| **Information Consumption** |  |  |  |  |  |  |  |  |  |  |  |
| **IC5** | 6.716* | .395 | 2.716 | 6.367 | .374* | 2.375 | 7.117* | .415 | 3.124 | 1 | 1 |
| **Socio-Economic Status** |  |  |  |  |  |  |  |  |  |  |  |
| **SES1** |  |  |  |  |  |  |  |  |  |  |  |
| **SES3** |  |  |  |  |  |  |  |  |  |  |  |
| **SES3** |  |  |  |  |  |  |  |  |  |  |  |
| **SES9** |  |  |  |  |  |  |  |  |  |  |  |

$$DV=dependent\mathrm{variable},$$

$$IVs=independent\mathrm{variables}$$

*The table records only the results of the regression analysis for predictors with statistically significant values of B coefficients $(Sig.\text{ }t<.05)$

Table A.14 Regression of the ”reputation of local politicians” variable

| **DV: SC6** | **Model 6.1** | | | **Model 6.2** | | | **Model 6.3** | | | | |
| --- | --- | --- | --- | --- | --- | --- | --- | --- | --- | --- | --- |
| **IVs:** | $F=1.212$ $(Sig.\text{ F}=.0297>.05)$ $Adjusted\text{ }R\text{ }Square=.103$ | | | $F=2.652$ $(Sig.\text{ F}=.003<.05)$ $Adjusted\text{ }R\text{ }Square=.292$ $Constant=3.898$ | | | $F=14.854$ $(N=112\text{ }Sig.\mathrm{F}\text{ }=.000<.05)$ $Adjusted\text{ }R\text{ }Square=.200$ $Constant=4.668$ | | | | |
|  | $R\text{ }Square\text{ }Change=.189$ $(F\text{ }Change=8.807;\text{ }Sig.\text{ }F\text{ }Change=.001<.05)$ | | | | | | $Durbin-Watson\text{ }=\text{ }1.9$ | | | *Collinearity Statistics* | |
| **Urbanism** | B | Beta | t | B | Beta | t | B | Beta | t | T | VIF |
| **U2** |  |  |  |  |  |  |  |  |  |  |  |
| **U3** |  |  |  |  |  |  |  |  |  |  |  |
| **U5** |  |  |  |  |  |  |  |  |  |  |  |
| **U11** |  |  |  |  |  |  |  |  |  |  |  |
| **U12** |  |  |  |  |  |  |  |  |  |  |  |
| **Information Consumption** |  |  |  |  |  |  |  |  |  |  |  |
| **IC2** |  |  |  | -1.919* | -.213 | -2.088 | -2.173* | -.241 | -2.836 | 1 | 1 |
| **IC4** |  |  |  |  |  |  |  |  |  |  |  |
| **IC8** |  |  |  |  |  |  |  |  |  |  |  |
| **IC9** |  |  |  |  |  |  |  |  |  |  |  |
| **Socio-Economic Status** |  |  |  |  |  |  |  |  |  |  |  |
| **SES1** |  |  |  |  |  |  |  |  |  |  |  |
| **SES2** |  |  |  |  |  |  |  |  |  |  |  |
| **SES4** |  |  |  |  |  |  |  |  |  |  |  |
| **SES5** |  |  |  |  |  |  |  |  |  |  |  |
| **SES8** |  |  |  | 4.841* | .420 | 4.209 | 4.545* | .386 | 4.542 | 1 | 1 |

$$DV=dependent\mathrm{variable},$$

$$IVs=independent\mathrm{variables}$$

*The table records only the results of the regression analysis for the predictors with statistically significant values of B coefficients $(Sig.\text{ }t<.05)$, The table for model 6.1. does not comprise information, because the model is not statistically significant $Sig.\mathrm{F}>.05$

Table A.16 Values of the standardized (Beta) regression coefficients, identified by regressing the variables that measure dimensions of social capital

| **Beta coefficients** | **Dependent variables** | | | | | |
| --- | --- | --- | --- | --- | --- | --- |
| **Independent variables** | SC1 | SC2 | SC3 | SC4 | SC5 | SC6 |
| **U1** |  |  |  | .183 |  |  |
| **U3** |  | .231 | .537 | -.107 |  |  |
| **U4** |  |  | -.124 | .880 |  |  |
| **U5** | -.260 |  |  |  |  |  |
| **U10** |  | -.277 |  | .069 |  |  |
| **U12** |  | .507 |  | .330 | .466 |  |
| **U13** |  |  | .197 |  |  |  |
| **U14/U1** |  | .187 |  |  |  |  |
| **IC1** | .456 |  |  |  |  |  |
| **IC 2** |  |  |  |  |  | -.241 |
| **IC 5** |  |  |  |  | .415 |  |
| **IC9/U15** |  | -.440 | -.283 | .092 |  |  |
| **SES8** |  |  |  |  |  | 4.542 |
