# Supplementary figures and images for: The Influence of Urbanism and Information Consumption on Political Dimensions of Social Capital: Exploratory Study of the Localities Adjacent to the Core City from Brașov Metropolitan Area, Romania
Source: PLoS One. 2016 Jan 25;11(1):e0144485. doi: 10.1371/journal.pone.0144485 (PMC4726559; doi:10.1371/journal.pone.0144485)

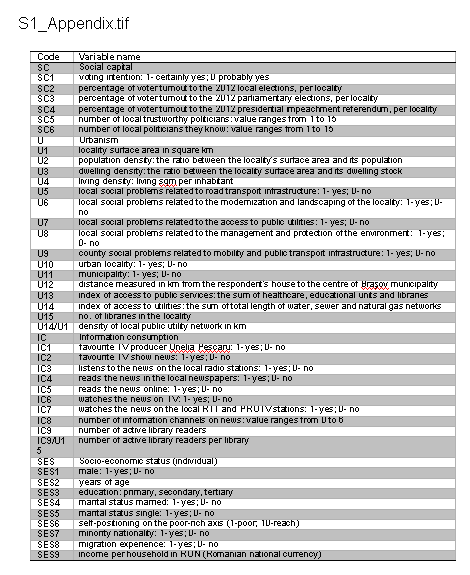

Supplement: S1 Appendix — (TIF) [file pone.0144485.s002.tif]

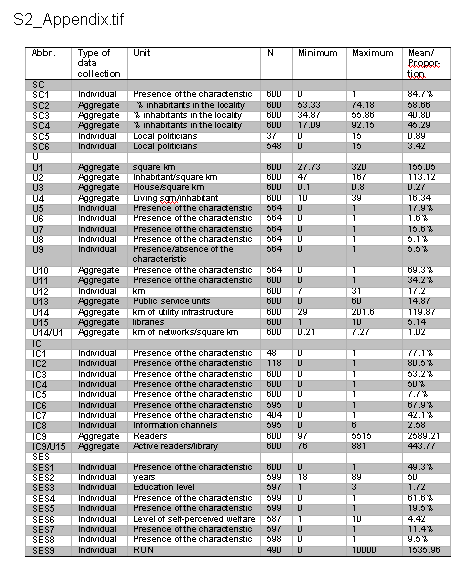

Supplement: S2 Appendix — (TIF) [file pone.0144485.s003.tif]

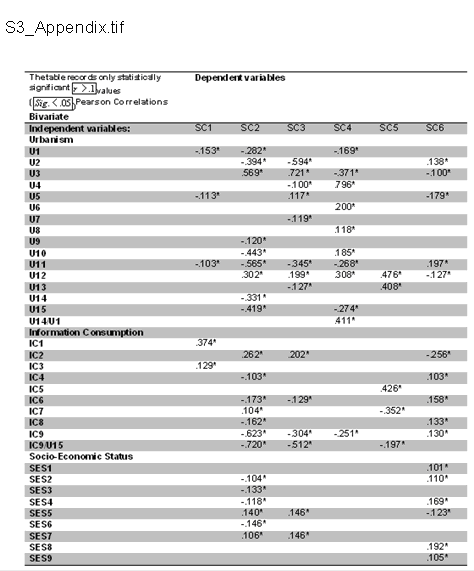

Supplement: S3 Appendix — (TIF) [file pone.0144485.s004.tif]
