# Supplementary material for: The Influence of Urbanism and Information Consumption on Political Dimensions of Social Capital: Exploratory Study of the Localities Adjacent to the Core City from Brașov Metropolitan Area, Romania
Source: PLoS One. 2016 Jan 25;11(1):e0144485. doi: 10.1371/journal.pone.0144485 (PMC4726559; doi:10.1371/journal.pone.0144485)
Supplement: S4 Appendix — (PDF) [file pone.0144485.s005.pdf]

S4_Appendix.pdf

Table A. Regression of U2 with U10. DV: U2	Model 1a				
IV:	$F = 13.217$ ($N = 593$ $Sig.F = .000 < .05$) $Adjusted\ R\ Square = .200$ $Constant = 124.411$				
	$Durbin-Watson = 1.8$			Collinearity Statistics	
Urbanism	B	Beta	t	T	VIF
U12	-.778*	-.148	-3.635	1	1

DV = dependent variable, IV = independent variable

U2 = population density, U12 = distance from the respondent residence to the center of Brailov municipality

*The table records only the results of the regression analysis for predictors with statistically significant values of B coefficients ($Sig.t < .05$)

Table B. Regression of U2 with U10.

DV: U2	Model 1b				
IV:	$F = .424$ ($N = 547$ $Sig.F = .514 > .05$) $Adjusted\ R\ Square = -.001$ $Constant = 408.958$				
	$Durbin-Watson = 1.9$			Collinearity Statistics	
Urbanism	B	Beta	t	T	VIF
U10					

DV = dependent variable, IV = independent variable

U2 = population density, U10 = urban administrative status of the locality *The table records only the results of the regression analysis for the predictors with statistically significant values of B coefficients. The table for model 1b does not comprise information, because the model is not statistically significant ($Sig.F > .05$)

Table C. Regression of U13 on U12.

DP: U13	Model 2a				
IV:	$F = 8.759$ ($N = 593$ $Sig.F = .000 < .05$) $Adjusted\ R\ Square = .444$ $Constant = -28.958$				
	$Durbin-Watson = 1.4$			Collinearity Statistics	
Urbanism	B	Beta	t	T	VIF
U12	2.549*	.669	21.871	1	1

DV = dependent variable, IV = independent variable

U13 = Index of access to public services, U12 = distance from the respondent residence to the center of Brailov municipality

*The table records only the results of the regression analysis for predictors with statistically significant values of B coefficients ($Sig.t < .05$)

Table D. Regression of U13 on U10.

DV: U13	Model 2b				
IV:	$F = 132.594$ ($N = 547$ $Sig.F = .000 < .05$) $Adjusted\ R\ Square = -1.89$ $Constant = 62130.285$				
	$Durbin-Watson = 1.3$			Collinearity Statistics	
Urbanism	B	Beta	t	T	VIF
U10	22.697*	.436	11.505	1	1

DV = dependent variable, IV = independent variable

U13 = Index of access to public services, U10 = urban administrative status of the locality

*The table records only the results of the regression analysis for predictors with statistically significant values of B coefficients $(Sig.t < .05)$

Table E. Regression of U14 on U12.

DV: U14	Model 3a				
IV:	$F = 6.144$ $(N = 593$ $Sig.F = .013 < .05)$ Adjusted R Square $= .009$ Constant $= 134.612$				
	$Durbin-Watson = 1.2$			Collinearity Statistics	
Urbanism	B	Beta	t	T	VIF
U12	-.857*	-.102	-2.483	1	1

DV = dependent variable, IV = independent variable

U13 = Index of access to public utilities, U12 = distance from the respondent residence to the center of Brasov municipality

*The table records only the results of the regression analysis for predictors with statistically significant values of B coefficients $(Sig.t < .05)$

Table F. Regression of U14 on U10.

DV: U14	Model 3b				
IV:	$F = 804.323$ $(N = 547$ $Sig.F = .000 < .05)$ Adjusted R Square $= -.587$ Constant $= 94412.982$				
	$Durbin-Watson = 1.9$			Collinearity Statistics	
Urbanism	B	Beta	t	T	VIF
U10	89.516*	.766	28.361	1	1

DV = dependent variable, IV = independent variable

U13 = Index of access to public utilities, U10 = urban administrative status of the locality

*The table records only the results of the regression analysis for predictors with statistically significant values of B coefficients $(Sig.t < .05)$
