# Supplementary material for: The Influence of Urbanism and Information Consumption on Political Dimensions of Social Capital: Exploratory Study of the Localities Adjacent to the Core City from Brașov Metropolitan Area, Romania
Source: PLoS One. 2016 Jan 25;11(1):e0144485. doi: 10.1371/journal.pone.0144485 (PMC4726559; doi:10.1371/journal.pone.0144485)
Supplement: S5 Appendix — (PDF) [file pone.0144485.s006.pdf]

DV: SC1	Model 1.1			Model 1.2			Model 1.3				
IVs:	F = 7.838 (Sig. F = .000 < 0.05			F = 4.147 (Sig. F = .003 < 0.05)			F = 8.759 (N = 47 Sig. F = .001 < .05)				
	Adjusted R Square = .340			Adjusted R Square = .321			Adjusted R Square = .254				
	Constant = .484			Constant = .439			Constant = .812				
	R Square Change = .033 (Sig. F Change = .585 > .05)						Durbin – Watson = 1.8			Collinearity Statistics	
Urbanism	B	Beta	t	B	Beta	t	B	Beta	t	T	VIF
U11											
U5				-.288*	-.288	-2.067	-.260*	-.260	-2.021	1	1
Information Consumption											
IC1	.264*	.544	8.048	.382*	.581	4.274	.264*	.456	3.541	1	1
Socio-Economic Status											
SES2											
SES3											
SES9											

DV = dependent variable, IVs = independent variables

SC1 = voting intention

*The table records only the results of the regression analysis for predictors with statistically significant values of B coefficients (Sig. t < .05)

Table B. Regression of SC2 (optimized model 2.3)

· DV: SC2	Model 2.1			Model 2.2			Model 2.3				
IVs:	F = 84.018			F = 56.074			F = 53.353				
	(Sig. F = .000 < .05)			(Sig. F = .000 < .05)			(N = 540 Sig. F = .000 < .05)				
	Adjusted R Square = .913			Adjusted R Square = .913			Adjusted R Square = .743				
	Constant = 48.835			Constant = 48.312			Constant = 53.353				
	R Square Change = .004						Durbin – Watson = 1.4			Collinearity Statistics	
	(F Change = .959; Sig. F Change = .444 > .05)										
Urbanism	B	Beta	t	B	Beta	t	B	Beta	t	T	VIF
U1											
U2											
U3	95.678*	.204	4.208	.009*	.067	.892	97.258*	.231	9.074	07	14
U5											
U10							-1.597*	-.177	-6.277	06	17
U11	19.990*	2.666	13.503	18.918*	2.523	11.780					
U12	.509*	.908	19.232	.503*	.989	17.630	.332*	.507	21.869	08	12
U14	-.035*	-.503	-3.449	-.035*	-.504	-3.379					
U15	3.402*	2.715	15.072	3.233*	2.580	13.235					

U14/U1							1.113*	.187	6.990	06	16
Information consumption											
CI2											
CI7											
CI8											
CI9	-.009*	-4.704	-15.989	-.009*	-4.464	-13.963					
CI9/U15							-.008*	-.440	-14.833	05	19
Socio-economic status											
SES2											
SES3				.480*	.078	2.113					
SES4											
SES5											
SES6											
SES7											
SES8				-.360*	.024	-.773					

DV = *dependent* variable, *IVs* = *independent* variables

SC2 = percentage of voter turnout to the 2012 local elections, per locality" variable

*The table records only the results of the regression analysis for predictors with statistically significan values of B coefficients ($Sig.t < .05$)

Table C. Regression of SC3 (optimized model 3.3).

DV: SC3	Model 3.1			Model 3.2			Model 3.3				
IVs:	$F = 29.841$ ($Sig.F = .000 < .05$) Adjusted R Square = .702; Constant = 39.329			$F = 23.141$ ($Sig.F = .000 < .05$) Adjusted R Square = .707; Constant = 34.241			$F = 245.581$ (N = [illegible] $Sig.$ = .000 < .05) Adjusted R Square = .665; Constant = 40.653				
	R Square Change = .012 (F Change = 1.557; $Sig.F$ Change = .205 > .05)						Durbin – Watson = 1.5			Collinearity Statistics	
Urbanism	B	Beta	t	B	Beta	t	B	Beta	t	T	VIF
U2	-.044*	-.375	-3.382	-.038*	-.324	-2.859					
U3	206.327*	.583	7.261	234.309*	.662	7.020	18146*	.537	15.406	05	22
U4											
U7	-1.530*	-.147	-2.684	-1.319*	-.127	-2.259	-1.321*	-.124	-5.193	1	1
U11	-2.667*	-.395	-1.929	-3.676*	-.544	-2.504					
U12											
U13	.041*	.315	2.185	.051*	.388	2.552	.032*	.197	7.336	08	13
Information Consumption											
CI2											
CI9	.001*	.383	1.903	.001*	.533	2.461					
CI9/U15							-.005*	-.283	-10.36	07	13

Socio-Economic Status											
SES5											
SES7											

DV = dependent variable, IVs = independent variables

SC3 = percentage of voter turnout to the 2012 parliamentary elections, per locality

*The table records only the results of the regression analysis for predictors with statistically significant values of B coefficients $(Sig.\,t < .05)$

Table D. Regression of SC4 (optimized model 4.3).

DV: SC4	Model 4.1			Model 4.2			Model 4.3				
IVs:	$F = 258158$ $(Sig.\,F = .000 < .05)$ Adjusted R Square $= .858$ Constant $= 9.230$			$F = 199142$ $(Sig.\,F = .000 < .05)$ Adjusted R Square $= .858$ Constant $= 9.370$			$F = 430.244$ $(N = 540,\ Sig.\,F = .000 < .05)$ Adjusted R Square $= .822$ Constant $= 1.414$				
	R Square Change $= .001$ $(F$ Change $= 1.197;$ $Sig.\,F$ Change $= .310 > .05)$						Durbin – Watson $= 1.5$			*Collinearity Statistics*	
Urbanism	B	Beta	t	B	Beta	t	B	Beta	t	T	VIF
U1	.039*	.355	9.055	.039*	.355	9.037	.019*	.183	6.324	04	26
U3	-122.018*	-.106	-4.816	-120.267*	-.104	-4.722	-117.340*	-.107	-4.948	07	15
U4	1.765*	.820*	32.642	1.768*	.821	32.660	1.889*	.880	39.740	06	16
U6											
U8											
U10	4.851*	.196	5.587	4.850*	.196	5.523	1.618*	.069	2.225	03	3
U11	-7.209*	-.318	-3.221	-6.999*	-.309	-3.121					
U12	.346*	.202	7.184	.341*	.199	6.935	.560*	.330	16.778	08	12
U15	-2.013*	-.538	-6.275	-2.029*	-.542	-6.313					
Information Consumption											
C8/U15							.004*	.092	3.850	05	18
C8	.003*	.507	3.145	.003*	.503	3.117					
Socio-Economic Status											
SES2											
SES3											
SES9											

DV = dependent variable, IVs = independent variables

SC4 = percentage of voter turnout to the 2012 presidential impeachment referendum, per locality

*The table records only the results of the regression analysis for predictors with statistically significant values of B coefficients $(Sig.\,t < .05)$

Table E. Regression of SC6 (optimized model 6.3).

DV: SC5	Model 5.1			Model 5.2			Model 5.3				
IVs:	$F = 7.733$<br>($Sig.\mathbf{F} = .001 < .05$)<br>$Adjusted\ R\ Square = .402$<br>$Constant = -3.873$			$F = 4.113$<br>($Sig.\mathbf{F} = .004 < .05$)<br>$Adjusted\ R\ Square = .384$<br>$Constant = -4.832$			$F = 11.293$<br>($N = 37\ Sig.\mathbf{F} = .000 < .05$)<br>$Adjusted\ R\ Square = .344$<br>$Constant = -3.288$				
	$R\ Square\ Change = .045$<br>($F\ Change = .728;\ Sig.\ F\ Change = .544 > .05$)						$Durbin-Watson = 2.4$			*Collinearity Statistics*	
Urbanism	B	Beta	t	B	Beta	t	B	Beta	t	T	VIF
U12	.309*	.510	2.814	.313	.516*	2.389	.261*	.466	3.507	1	1
U13											
Information Consumption											
IC5	6.716*	.395	2.716	6.367	.374*	2.375	7.117*	.415	3.124	1	1
Socio-Economic Status											
SES1											
SES3											
SES8											
SES9											

DV = dependent variable, IVs = independent variables

SC5 = trust in local politicians

*The table records only the results of the regression analysis for predictors with statistically significant values of B coefficients ($Sig.\ t < .05$)

Table F. The regression of SC6 (optimized model 6.3).

DV: SC6	Model 6.1			Model 6.2			Model 6.3				
IVs:	$F = 1.212$<br>($Sig.\mathbf{F} = 0.297 > .05$)<br>$Adjusted\ R\ Square = .103$			$F = 2.452$<br>($Sig.\mathbf{F} = .003 < .05$)<br>$Adjusted\ R\ Square = .292$<br>$Constant = 3.898$			$F = 14.854$<br>($N = 112\ Sig.\mathbf{F} = .000 < .05$)<br>$Adjusted\ R\ Square = .200$<br>$Constant = 4.468$				
	$R\ Square\ Change = .189$<br>($F\ Change = 8.807;\ Sig.\ F\ Change = .001 < .05$)						$Durbin-Watson = 1.9$			*Collinearity Statistics*	
Urbanism	B	Beta	t	B	Beta	t	B	Beta	t	T	VIF
U2											
U3											
U5											
U11											
U12											
Information Consumption											
IC2				-1.919*	-.213	-2.088	-2.173*	-.241	-2.836	1	1
IC4											
IC8											
IC9											
Socio-Economic Status											
SES1											
SES2											

SES4								
SES5								
SES8	4.841*	.420	4.209	4.545*	.386	4.542	1	1

DV = *dependent* variable, *IVs* = *independent* variables

SC6 = notoriety of local politicians

*The table records only the results of the regression analysis for the predictors with statistically significant values of B coefficients ($Sig. t < .05$). The table for model 6.1. does not comprise information, because the model is not statistically significant

$Sig. F > .05$
