# Supplementary material for: The Influence of Urbanism and Information Consumption on Political Dimensions of Social Capital: Exploratory Study of the Localities Adjacent to the Core City from Brașov Metropolitan Area, Romania
Source: PLoS One. 2016 Jan 25;11(1):e0144485. doi: 10.1371/journal.pone.0144485 (PMC4726559; doi:10.1371/journal.pone.0144485)
Supplement: S1 Aprouval — (DOC) [file pone.0144485.s007.doc]

Transilvania University Ethical Commision Aprouval.doc

*Translation from Romanian*

**“Transilvania” University from Brasov**

**Faculty of Medicine**

Brasov, str. Nicolae Balcescu, nr. 56

Tel/Fax: +40 268 412185 – [www.unitbv.ro](http://www.unitbv.ro/)

Transilvania University from Brasov is a higher education institution whose aims include training specialists, career development, evolution of knowledge and research, observing the rule of law and human rights.

The University respects the dignity of all and each member and promotes academic integrity. Its members are committed to help develop democracy and social prosperity, observing the University Charter, the ethics and deontological code and the applicable laws in force.

The Code regulates and promotes moral values, such as: academic freedom, personal autonomy, justice and equity, merit, professionalism, honesty and intellectual fairness, transparency, respect and tolerance, responsibility, kindness and care. The members of the academic community and the students have the obligation to comply with the deontological codes, the patient law, the labour code and all other provisions that regulate the professional and educational activity and research.

In line with the current international norms, an ethics committee has been established within the Faculty of Medicine of Transilvania University from Brasov (consisting of faculty members valued for their moral integrity and professionalism, both in their educational activity and their research), a committee that monitors the manner in which the main ethical criteria of medical scientific research are observed, in compliance with the regulations of the Declaration of Helsinki, adopted in 1964 and amended in 1975 Tokyo, 1983 Venice, 1989 Hong Kong, 1996 Sommerset West, 2000 Edinburgh, 2002 Washington, 2004 Tokyo, 2008 Seoul.

The faculty of Transilvania University from Brasov ensure the performance of medical research in full compliance with the principles of bioethics, supporting the embrace of a good practice in research. Educational and institutional benchmarks are provided concerning the assimilation and implementation of bioethical principles for initiating, performing and publishing clinical trials, animal studies and drug studies. The use of laboratory animals during the teaching process or during the medical research shall comply with the legal regulations in force.

Mr. Prof. Univ. Dr. Coman Claudiu, director of the Department of Social Sciences and Communication, has submitted to the ethics committee the approval of the data retrieval procedure for the purpose of conducting secondary analyses, from the research database of ”Socio-Political Situation of Brasov County”.

The subjects’ consent to reply to questions has been obtained in an informed manner. The responders were verbally informed by the interview operators regarding the purpose of the research, providing insurance with respect to their confidentiality and privacy. The responders were also informed regarding their right to decide whether or not to participate to the study, in view of the lack of any financial compensation, or of any sanctions in case of refusal, as well as regarding their right, at any time throughout the interview, to refrain from answering specific questions, or to rescind their participation entirely. The responders’ acceptance or refusal to participate to the study was documented in the sampling files (with a 10% share of non-replies).

The research shall also comply with the following regulations:

- Law no. 206/2004 on good conduct in scientific research, technological development and innovation, with subsequent amendments and completions
- The Nuremberg Code

The team conducting this study is coordinated by Prof. Univ. Dr. Coman Claudiu and is listed in the request to the application submitted to the ethics committee.

The training of the study team members for the purpose of complying with the patient’s rights was performed by Prof. Univ. Dr. Coman Claudiu, as main investigator.

President of the Ethics Committee for Scientific Research

of the Faculty of Medicine

Prof. Univ. Dr. Liliana ROGOZEA

(*illegible signature*)

Members:

Prof. Univ. Dr. Mihaela Gheorghe

(*illegible signature*)

Prof. Univ. Dr. Jr. Adrian Manea

Priest: Prof. Univ. Dr. Ovidiu Mocanu

(*illegible signature*)

As. Univ. Drd. Florin Leasu

(*illegible signature*)
