# Supplementary material for: The Influence of Urbanism and Information Consumption on Political Dimensions of Social Capital: Exploratory Study of the Localities Adjacent to the Core City from Brașov Metropolitan Area, Romania
Source: PLoS One. 2016 Jan 25;11(1):e0144485. doi: 10.1371/journal.pone.0144485 (PMC4726559; doi:10.1371/journal.pone.0144485)
Supplement: S1 Survey — (DOC) [file pone.0144485.s008.doc]

survey_EN_PlosOne.doc

***(f) If local elections were to be held next Sunday, would you go to vote?***

| Certainly yes | Probably yes | Probably no | Certainly no | I don't know/I do not answer |
| --- | --- | --- | --- | --- |
|  |  |  |  |  |

(q6) ***What are the main local problems that should be addressed with priority?***

(q6a) (first option)………………………………………………………………………………………………………………………

(q15) **How much trust do you have in …?**

|  | | A great deal | Fair amount | Little | Very little | I don't know him/her | I don't know/I do not answer |
| --- | --- | --- | --- | --- | --- | --- | --- |
| q15.1 | Donțu Mihai |  |  |  |  |  |  |
| q15.2 | Grapă Sebastian |  |  |  |  |  |  |
| q15.3 | Rasaliu Marian |  |  |  |  |  |  |
| q15.4 | Puchianu Ioan |  |  |  |  |  |  |
| q15.5 | Zanfir Dan |  |  |  |  |  |  |
| q15.6 | Ochi Ioan |  |  |  |  |  |  |
| q15.7 | Niță Constantin |  |  |  |  |  |  |
| q15.8 | Chiriac Viorel |  |  |  |  |  |  |
| q15.9 | Gabor Gheorghe |  |  |  |  |  |  |
| q15.10 | Drăghici Cristi |  |  |  |  |  |  |
| q15.11 | Pascu Mihai |  |  |  |  |  |  |
| q15.12 | Kovacs Attila |  |  |  |  |  |  |
| q15.13 | Fliundra Ionel |  |  |  |  |  |  |
| q15.14 | Danu Aurelian |  |  |  |  |  |  |
| q15.15 | Vatafu Valer |  |  |  |  |  |  |

***(m) Do you follow the news on any of the following information channels?***

|  |  | Yes | No | I don't know/I do not answer |
| --- | --- | --- | --- | --- |
| (m_a) | Radio |  |  |  |
| (m_b) | Local printed press |  |  |  |
| (m_c) | Internet |  |  |  |
| (m_d) | Television |  |  |  |

(m1) ***What local newspaper do you read most frequently to find out the news?***

| …………………………………………………………………………. |  |
| --- | --- |
| I do not read local news in the local newspapers |  |
| I don't know/I do not answer |  |

(m2) ***Do you read local news from newspapers distributed for free?***

| Yes |  | No |  | I don't know/I do not answer |  |
| --- | --- | --- | --- | --- | --- |

***(m3)*** Do you read local news from local press on online news portals?

| Yes |  | No |  | I don't know/I do not answer |  |
| --- | --- | --- | --- | --- | --- |

***(m6) What local radio station do you listen to most frequently to find out local news?***

| …………………………………………………………………………. |  |
| --- | --- |
| I do not listen to local news on local radio stations |  |
| I don't know/I do not answer |  |

***(m6)*** What central radio station do you listen to most frequently to find out local news?

| …………………………………………………………………………. |  |
| --- | --- |
| I do not listen to local news on central radio stations |  |
| I don't know/I do not answer |  |

(m11) ***What is your favourite TV show?***

| …………………………………………………………………………. |  |
| --- | --- |
| I do not have a favourite TV show |  |
| I don't know/I do not answer |  |

(m11) ***What is your favourite TV producer?***

| …………………………………………………………………………. |  |
| --- | --- |
| I do not have a favourite TV producer |  |
| I don't know/I do not answer |  |

***(m13)*** What local TV station do you watch most frequently to find out local news?

| …………………………………………………………………………. |  |
| --- | --- |
| I do not watch local news on local TV stations |  |
| I don't know/I do not answer |  |

(sex) ***Please note down the sex of the respondent:***

| male |  | female |  |
| --- | --- | --- | --- |

(age) ***How many years have you turned?***

| …………………. years |  | I don't know/I do not answer |  |
| --- | --- | --- | --- |

(school) ***What is the last graduated school?***

| Primary/secondary |  |
| --- | --- |
| Highschool/further education/vocational technical school |  |
| University/Masters/Post-university |  |
| I don't know/I do not answer |  |

(marital_status) ***What is you marital status?***

| Married |  | Consensual union |  |
| --- | --- | --- | --- |
| Divorced |  | Single |  |
| Widow |  | Other situations ..................................... |  |
| Separated, without divorce |  | I don't know/I do not answer |  |

(scale) ***On a scale from 1 to 10 (1 meaning poor and 10 meaning rich), where do you position yourself?***

| 1 | 2 | 3 | 4 | 5 | 6 | 7 | 8 | 9 | 10 |
| --- | --- | --- | --- | --- | --- | --- | --- | --- | --- |
| poor |  |  |  |  |  |  |  |  | rich |
|  |  |  |  |  |  |  |  |  |  |

(income) ***What is the net income of your family?***

| ………………………………………………………………………….RON |  |
| --- | --- |
| We have no income |  |
| I don't know/I do not answer |  |

(nationality) ***What is your nationality?***

| …………………………………………………………………………. |  |
| --- | --- |
| I don't know/I do not answer |  |

***(d10) Is anyone from your family currently working abroad?***

| Yes |  | No |  | I don't know/I do not answer |  |
| --- | --- | --- | --- | --- | --- |

(local) ***Please note down the locality:***

| Săcele |  | Cristian |  | Racoș |  | Drăguș |  |
| --- | --- | --- | --- | --- | --- | --- | --- |
| Bod |  | Holbav |  | Feldioara |  | Sâmbăta de Sus |  |
| Sânpetru |  | Vulcan |  | Hoghiz |  | Victoria |  |
| Hărman |  | Poiana Mărului |  | Ungra |  | Ucea |  |
| Vama Buzăului |  | Predeal |  | Ticușu |  | Făgăraș |  |
| Teliu |  | Ghimbav |  | Comana |  | Mândra |  |
| Budila |  | Codlea |  | Părău |  | Șinca Veche |  |
| Tărlungeni |  | Bunești |  | Jibert |  | Șinca Nouă |  |
| Prejmer |  | Homorod |  | Șoarș |  | Recea |  |
| Hălchiu |  | Cața |  | Cincu |  | Hârseni |  |
| Zărnești |  | Rupea |  | Dumbrăvița |  | Lisa |  |
| Râșnov |  | Măieruș |  | Crizbav |  | Beclean |  |
| Fundata |  | Apața |  | Șercaia |  | Brașov |  |
| Moieciu |  | Ormeniș |  | Voila |  |  | |
| Bran |  | Augustin |  | Viștea |  |

***(local_type) Please note down that type of locality:***

| Rural |  | Small urban |  | Municipality |  |
| --- | --- | --- | --- | --- | --- |
